# Melanin‐Like Nanomaterials for Advanced Biomedical Applications: A Versatile Platform with Extraordinary Promise

**DOI:** 10.1002/advs.201903129

**Published:** 2020-02-07

**Authors:** Heng Liu, Youyuan Yang, Yu Liu, Jingjing Pan, Junqing Wang, Fengyuan Man, Weiguo Zhang, Gang Liu

**Affiliations:** ^1^ Department of Radiology PLA Rocket Force Characteristic Medical Center Beijing 100088 China; ^2^ Department of Radiology Daping Hospital Army Medical University Chongqing 400042 China; ^3^ Department of Ultrasound The First Affiliated Hospital Army Medical University Chongqing 400038 China; ^4^ School of Pharmaceutical Sciences (Shenzhen) Sun Yat‐sen University Guangzhou 510275 China; ^5^ Chongqing Clinical Research Center for Imaging and Nuclear Medicine Chongqing 400042 China; ^6^ Laboratory of Molecular Vaccinology and Molecular Diagnostics & Center for Molecular Imaging and Translational Medicine School of Public Health Xiamen University Xiamen 361102 China

**Keywords:** biomedicine, melanins, nanomaterials, polydopamine, theranostics

## Abstract

Developing efficient, sustainable, and biocompatible high‐tech nanoplatforms derived from naturally existing components in living organisms is highly beneficial for diverse advanced biomedical applications. Melanins are nontoxic natural biopolymers owning widespread distribution in various biosystems, possessing fascinating physicochemical properties and playing significant physiological roles. The multifunctionality together with intrinsic biocompatibility renders bioinspired melanin‐like nanomaterials considerably promising as a versatile and powerful nanoplatform with broad bioapplication prospects. This panoramic Review starts with an overview of the fundamental physicochemical properties, preparation methods, and polymerization mechanisms of melanins. A systematical and well‐bedded description of recent advancements of melanin‐like nanomaterials regarding diverse biomedical applications is then given, mainly focusing on biological imaging, photothermal therapy, drug delivery for tumor treatment, and other emerging biomedicine‐related implementations. Finally, current challenges toward clinical translation with an emphasis on innovative design strategies and future striving directions are rationally discussed. This comprehensive and detailed Review provides a deep understanding of the current research status of melanin‐like nanomaterials and is expected to motivate further optimization of the design of novel tailorable and marketable multifunctional nanoplatforms in biomedicine.

## Graphical abstract

Melanins are nontoxic natural biopolymers owning widespread distribution in various biosystems and play significant physiological roles. The fascinating physicochemical properties of melanins make bioinspired melanin‐like nanomaterials considerably promising as a versatile and powerful nanoplatform with broad bioapplication prospects. This panoramic Review is expected to bolster the understanding of melanin‐derived nanomaterials and motivate further optimization design of tailorable and marketable multifunctional nanoplatforms in biomedicine.

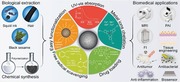

## 1 Introduction

Rapid advances in nanotechnology and biomedicine offer great opportunities in the field of nanomedicine. Nature is an immense treasury for scientists, which provides inexhaustible sources of inspiration to design and construct multifunctional biomaterials and devices with extraordinary properties. Developing efficient, sustainable, and biocompatible high‐tech nanoplatforms derived from naturally existing components in living organisms is highly beneficial for diverse advanced biomedical applications, arising from their competence to decomposition into nontoxic metabolites during retention in vivo and thus minimized adverse effects.^1^ This is crucial to guarantee their biosafety in vivo. Melanins are ubiquitous natural biopolymers owning widespread distribution in various parts of living organisms, such as hair, skin, mucous membranes, brain medulla, and eyes.^2^ They are well known to participate in a broad scope of biochemical pathways and biological functions owing to their intriguing physicochemical properties,^3^ such as broadband ultraviolet–visible (UV–vis) absorption,^4^ intrinsic photoacoustic property,^5^ coloration,^6^ photo and electromagnetic radiation protection,^7^ antioxidation,^8^ free radical scavenging,^9^ ultrafast thermal relaxation,^10^ high chelating capability to metal ions,^11^ nonradiative relaxation of photo‐induced electron states,^3^ some nervous system involvement,^12^ and so forth. These fascinating physicochemical properties are closely related to the unique chemical structures and composition of melanins. More interestingly, melanins possess inherent biocompatibility and biodegradability, simple and mild preparation procedures, and easy functionalization. Accordingly, these prominent features make bioinspired melanin‐like nanoparticles (MelNPs) considerably promising as a versatile and powerful nanoplatform for diverse biomedical applications.^13^ In recent years, much significant research developments in various aspects has been achieved. Several excellent Reviews have been published with a main emphasis on polydopamine (PDA) as a versatile surface coating material in biomedicine, new energy and environment implementation.^14^ For instance, Cheng et al. mainly highlighted the achievements of PDA platforms for surface modification and nanomedicine.[qv: 14g] Huang's group summarized the biomedical applications of melanin/PDA‐based nanomaterials.[qv: 14h] Thus far, however, considerable length in these existing literatures are about PDA‐based surface modification, which cannot represent the melanin‐like nanomaterials. And they do not provide systematical, comprehensive, and well‐bedded aspects of MelNPs regarding diverse biomedical applications. A detailed and pertinent panoramic view of the state‐of‐the‐art progress of MelNPs in biomedicine is urgently demanded to grasp the research status and striving directions of MelNPs well, which is instructive to promote further development and clinical translation.

Different from previous reviews on this topic, this panoramic Review starts with an overview of the fundamental physicochemical properties, preparation methods, and polymerization mechanisms of melanins. It then gives a systematical and well‐organized description of recent advancements of melanin‐like nanomaterials regarding diverse biomedical applications during the past decade, including biological imaging (e.g., fluorescence, photoacoustic, magnetic resonance, nuclear medical, and multi‐modal imaging), photothermal therapy (PTT), drug delivery for tumor treatment (e.g., pH‐responsive drug release, monotherapy, and bi‐modal synergistic therapy), and other applications (e.g., antioxidative and anti‐inflammatory therapy, wound healing and medical bioadhesives, antibacterial infection, irradiation protection, neuroprotection in Parkinson's disease, and biosensing). At the end, current challenges associated with clinical translation with an emphasis on innovative strategies, striving directions and future prospects are discussed rationally. As PDA‐based surface modification have been summarized and discussed in many previous Reviews, they are beyond the scope of this article and are not presented in unnecessary details. This comprehensive and pertinent Review is expected to deepen our comprehending of the current research status of melanin‐like nanomaterials and motivate further optimization design of tailorable and marketable multifunctional nanoplatforms in biomedicine.

## 2 Definition, Preparation Methods, and Polymerization Mechanisms

Although the chemical structures of melanins have been continuously investigated via various advanced analytical characterization techniques, there is still no unified definition due to the rich diversity in the origin, size, color, and function of melanins. To date, the commonly acknowledged definition of heterogeneous melanins in the academic world is “Melanins are mostly biopolymers formed from phenolic compounds by polymerization via quinones.”^15^ On the basis of precursor molecules in biosynthesis and elements in the composition, melanins are roughly categorized into three main types, namely brown‐black eumelanin, yellow‐reddish pheomelanin, and allomelanin.^13^ In nature, eumelanin and pheomelanin are derived from tyrosine precursor, and their main difference is whether contain elemental sulfur or not. Allomelanin is generally derived from 1,3,6,8‐tetrahydroxynaphthalene precursor, basically in absence of nitrogen and sulfur.^16^

Natural melanin granules with spherical or elliptical shapes have been obtained by extraction and purification from biological sources (e.g., cuttlefish ink,^17^ black sesame seeds,^18^ human hair^19^). However, there is a lack of widely accepted standardized extraction procedures yet, and current extraction and purification techniques are sometimes more complicated than artificial synthetic approaches. Moreover, due to little information on the exact chemical structures of melanins, it is difficult to determine whether all characteristics of natural pigments were well preserved following these extraction procedures. From another point of view, it is expected rationally that artificial synthetic melanins are of great value. PDA nanoparticles (PDA NPs), as the name implies, are nanostructures produced by oxidation–polymerization of dopamine monomers. Owing to the same precursor molecules and similar oxidation–polymerization processes in PDA NPs and eumelanin, PDA NPs are well regarded as a bioinspired synthetic analogue of naturally occurring eumelanin for simplified and reliable interpretation of the structure–property relationships.^20^ Recently, PDA NPs have been widely explored as melanin‐like nanomaterials for biophysical investigations and biomedical applications.

Artificial PDA NPs are usually fabricated by three common approaches, including solution oxidation, enzymatic oxidation and electropolymerization.^13^ Among them, solution oxidation method represents the most frequently used protocol, due to its convenient and mild preparation process without requiring complicated instruments and harsh reaction conditions.[qv: 14f] The dopamine monomers are oxidized and spontaneously self‐polymerize to crosslinked polymers under alkaline and aerobic conditions. In enzymatic oxidation method, precursor molecules such as phenol derivatives, phenolic compounds, and aromatic amines are usually polymerized in the presence of enzymes (e.g., tyrosinase (Tyr) enzyme, laccase, urease). The enzyme‐catalyzed strategy possesses relatively complicated processes while higher efficiency without waste production.^21^ As it is inspired by the biosynthesis of melanins in biosystems, the properties of the products are similar to naturally occurring melanin granules. For electropolymerization method, PDA are usually obtained by direct deposition on the surface of electrically conductive materials in a simple and effective manner.^22^ In comparison with solution oxidation method, this approach owns higher dopamine utilization and deposition rate. Importantly, the thickness of electropolymerized dopamine can be precisely controlled. By adjusting a variety of reaction parameters (e.g., pH and buffer solution, reaction time and temperature, oxidants, monomer and reactant concentration, amphiphilic surfactants), melanin‐like nanomaterials can be fabricated in various types of nanostructures, including but not limited to nanoparticles, hollow capsules, nanofibers, nanotubes, microfilms, dodecahedral hollow nanocontainers, flowerlike hierarchical nanostructures as well as core@shell nanostructures.[qv: 14g] Moreover, the morphology, size, yield and properties of the products can be well controlled and tuned.

Despite that a variety of considerably mature approaches have been well established for controllable synthesis of MelNPs, the related polymerization mechanisms and exact chemical structures of MelNPs remain elusive as yet. This is ascribed to abundant chemical reaction sites on precursor molecules, complicated oxidation–reduction reactions and a series of intermediate products involved in the polymerization process. The amorphous character, insolubility, and opaque nature of natural melanins also partly impede their accurate structure determination. The possible polymerization mechanism is considered to be similar to that of the biosynthesis pathway of eumelanin in living organisms. Several presumptive analytical models have been proposed for tentative elucidation, such as covalent bonding mode,^23^ and covalent mode in combination with noncovalent self‐assembly.^24^ Taking into account that the polymerization mechanisms of MelNPs have been summarized and discussed in many previous Reviews,[qv: 13,14g,20,25] they are beyond the scope of this section and are not presented in unnecessary details. Furthermore, based on theoretical and experimental methods, enormous efforts have also been directed toward understanding the structure–property–function relationship of MelNPs.^20^

## 3 Fundamental Physicochemical Properties

### 3.1 Broadband UV–Vis Absorption

Both natural eumelanin and synthetic MelNPs exhibit broadband monotonic UV–vis absorption spectra, which appears more like inorganic materials rather than organic. This broad featureless absorption profile is indeed in accord with amorphous banded semiconductors, which can be elucidated by the chemical‐ and geometric‐disorder models, scattering effects, and excitonic effects. As MelNPs can effectively absorb optical energy and transfer it into heat, they are highly attractive to serve as endogenous photoacoustic contrast agents and photothermal conversion agents.

### 3.2 Metal Ions Chelation Ability

Melanins can act as both metal reservoirs and metal sinks in vivo. The binding sites, binding capacity, and their relative affinity to metal ions have been well established. The interaction is concluded to depend on types of metal ions, amounts of metal ions binding sites, and reduced/oxidized state of catechol moieties. MelNPs exhibit high binding affinity toward various metal ions, attributed to the presence of abundant sites for metal coordination, including amine, carboxy, *o*‐quinone, semiquinone, phenolic groups, and nitrogen atoms on their surface.^11^ Inspired by this, MelNPs have been extensively employed as building blocks for chelating various paramagnetic metal ions (e.g., gadolinium,^26^ manganese,^27^ and iron^28^) as MR contrast agents, without requiring additional extrinsic chelators.

### 3.3 Drug Binding Capacity

MelNPs have plentiful π‐conjugated structures on their surface (e.g., dihydroxyindole, indolequinone), making them powerful to bind with various drugs with aromatic structures via π–π stacking, hydrogen bonding interaction, and van der Waals interaction.^29^ The drugs can be also conjugated onto the surface of MelNPs by chemical bonding,^30^ or encapsulated within the polymer matrix of MelNPs.^31^ Hence, MelNPs represent a promising drug nanocarrier candidate for drug delivery.[qv: 14e] These small molecule drugs include but not limit to chemotherapeutics (doxorubicin (DOX), sorafenib, camptothecin, paclitaxel, cisplatin, Pt(||) metallacycle, bortezomib, sanguinarine (SAN)), photosensitizers (chlorin e6, indocyanine green, pheophorbide‐a, zinc phthalocyanine), antibiotics (rifampicin, tetracycline hydrochloride, ciprofloxacin), angiogenic agents (dimethyloxalylglycine), and anti‐inflammatory drugs (aspirin).

### 3.4 High Chemical Reactivity and Easy Functionalization

MelNPs have abundant chemically active functional groups (e.g., catechol, *o*‐quinone, amine, and imine) on their surface. These groups allow the formation of covalent bonds with various with nucleophilic thiol‐ and amino‐containing molecules by means of Michael addition (with thiol groups), Schiff base reaction (with amine groups) and coordinative interactions.^23^ Aryl–aryl covalent bonding among units as well as conjugates with other molecules can be also generated by these reactions. Besides, the amino groups on the surface of MelNPs can also react with carboxyl groups containing molecules via a conventional carbodiimide reaction. This endows MelNPs chemical versatility with a myriad of substrates and feasibility for adding functionality.

### 3.5 Antioxidation and Free Radical Scavenging Ability

The defensive role of melanin in human skin against ultraviolet radiation induced damage is thought to be involved in blocking and scavenging free radicals produced by ultraviolet radiation.^32^ More Recently, this property was also confirmed by artificial allomelanin nanoparticles.^33^ Owing to the abundant reductive functional groups (e.g., catechol, amine, and imine), MelNPs provide potent and broad scavenging activities against multiple reactive oxygen and nitrogen species (RONS), such as hydrogen peroxide, hydroxyl radical, nitrogen monoxide radical, superoxide anion, and peroxynitrite.^34^ The unique radical scavenging ability of MelNPs render them promising as high‐performance antioxidative platforms.

### 3.6 Stability

Evidence suggests that the particle size and zeta potential of MelNPs vary in solutions with different pH and ionic strength, which is believed to be related to the repulsion forces and protonation status, respectively.^35^, ^133^ Appropriate surface modification can endow the MelNPs solution with excellent colloidal dispersion stability in various physiological media.^36^ Both natural sepia melanin and synthetic MelNPs exhibit a hydrogen peroxide‐responsive decomposition behavior, confirmed by optical absorption measurement and transmission electron microscopy.^36^, ^37^ This chemical degradation pathway can minimize the poor biodegradation issue of many other nanomaterials. The high photothermal stability of both natural melanin and synthetic MelNPs has been verified via cyclic‐irradiation assay in many investigations.^36^, ^38^ For instance, no significant decrease in temperature variation was observed over five laser irradiation cycles,[qv: 36a] and no obvious optical absorption and nanostructure morphology changes was observed after longstanding laser irradiation (2 W cm^−2^, 1 h).[qv: 36b] This suggests great potential of MelNPs for in vivo PTT applications.

## 4 Biomedical Applications

As MelNPs are biopolymers derived from natural sources, they show excellent biocompatibility and negligible toxicity. They are supposed to be in absence of adverse effects including cytotoxicity when cocultured with cells in vitro, and antigenic response when administration into living organisms. A high dosage of melanin up to 100 mg kg^−1^ body weight did not show any noticeable side effect on mice within 30 days.^39^ Furthermore, numerous evidences indicate the excellent hemocompatibility of MelNPs produced by biological extraction and chemical synthesis, confirmed by hemolytic test and blood routine analysis.[qv: 17,36a] The satisfactory biocompatibility and hemocompatibility of MelNPs make them suitable for biomedical applications.

**Figure 1:**
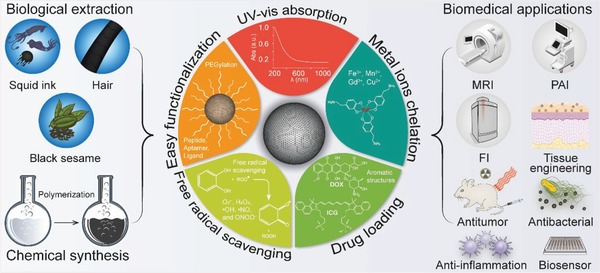
Schematic illustration of the synthesis, property, and biomedical applications of multifunctional MelNPs.

As aforementioned, the fascinating physicochemical properties of MelNPs make them highly promising as a simple and versatile nanoplatform for diverse biomedical applications, including bioimaging (e.g., fluorescence, photoacoustic, magnetic resonance, and multi‐modal imaging), photothermal therapy, drug delivery for tumor treatment (e.g., pH‐responsive drug release, monotherapy, bi‐modal synergistic therapy), antioxidative and anti‐inflammatory therapy, tissue engineering and medical bioadhesives, antibacterial infection, irradiation protection, neuroprotection in Parkinson's disease, and biosensing. The following section will provide systematical and well‐bedded description of recent advancements of melanin‐like nanomaterials regarding diverse biomedical applications during the past decade. Some representative studies will be presented. As PDA as a surface coating material has been summarized in many previous Reviews,[qv: 13,14g,20,25] they are beyond the scope of this section and are not presented in unnecessary details. Schematic illustration of the synthesis, property, and biomedical applications of multifunctional MelNPs are shown in **Figure** **1**.

### 4.1 Biological Imaging

#### 4.1.1 Fluorescence Imaging

**Figure 2:**
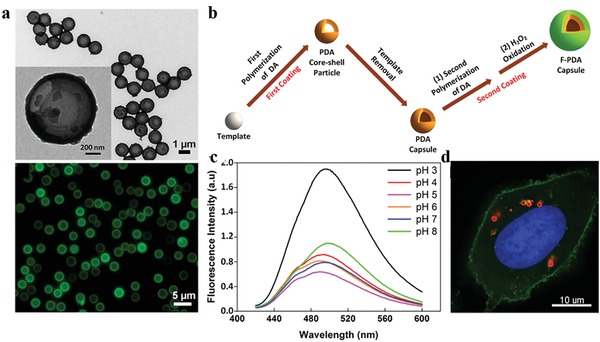
Engineering fluorescent PDA capsules for cell fluorescence imaging. a) TEM (upper) and fluorescence (lower) images of F‐PDA capsules. b) Schematic diagram of the synthesis procedure of F‐PDA capsules. c) Normalized fluorescence emission spectra of F‐PDA capsules under various pH conditions. d) Fluorescence images of F‐PDA capsules (red) internalized HeLa cells. Green, cell membrane; blue, nuclei. Reproduced with permission.^41^ Copyright 2014, American Chemical Society.

Recently, biocompatible fluorescent organic nanoparticles (FONs) have drawn considerable attention for fluorescence imaging applications. Compared with conventional small molecule organic dyes and fluorescent inorganic nanoparticles, they possess superior optical properties, lower toxicity, and better biodegradability. Due to the excellent biocompatibility and unique physicochemical properties, melanin‐like fluorescent nanomaterials have received considerable interest for molecular imaging in recent years. For instance, Zhang et al. first presented water soluble PDA‐based FONs with strong and tunable photoluminescence, which were facilely prepared by the simple one‐pot oxidation of PDA NPs using concentrated hydrogen peroxide.^40^ Compared with conventional methods for fabrication of FONs, this approach simplifies the synthesis procedures, and does not require hypertoxic chemical agents and tedious postmodification. After internalization by NIH‐3T3 cells, PDA FONs exhibited green and green‐yellow fluorescence under 405 and 458 nm laser excitation, respectively. This excitation wavelength‐dependent fluorescent behavior was resulting from the wide size distribution of PDA FONs. Based on this oxidative strategy, fluorescent PDA (F‐PDA) capsules were subsequently synthesized via the first dopamine polymerization on template particles, subsequent template core removal, and the second dopamine polymerization in the presence of hydrogen peroxide (**Figure** **2**).^41^ The fluorescence property was supposed to arise from the formation of small fluorescent species during the oxidative treatment by hydrogen peroxide. The size and morphology of F‐PDA capsules were well controlled by adopting different‐sized inorganic and organic template particles. The resultant F‐PDA capsules exhibited pH‐dependent fluorescence signals (Ex/Em = 400/480 nm), with the strongest signal at pH = 3. After incubation with HeLa cells for 24 h, the internalized F‐PDA capsules with spherical morphology were readily visualized using fluorescence microscopy.

In addition to the oxidation procedure, some convenient, green, and effective approaches have also been explored for obtaining PDA‐based FONs. For instance, polyethylenimine (PEI)‐PDA FONs were facilely prepared via self‐polymerization of dopamine in the presence of amino‐abundant polyethylenimine.^42^ The synthetic procedure was carried out under air atmosphere and room temperature, without the assistance of additional catalysts and initiators. As the excitation wavelength varied in the range of 340–480 nm, the as‐prepared PEI‐PDA FONs exhibited altered fluorescence intensity, with a fixed emission wavelength at 526 nm. Encouragingly, they demonstrated satisfactory results for cell fluorescence imaging even at a concentration as low as 10 µg mL^−1^. Subsequently, novel polymer‐FONs were strategically designed, combining reversible addition–fragmentation chain transfer polymerization and aforementioned PEI‐PDA FONs.^43^ The chemical compositions and functional groups of the final polymer‐FONs could be well adjusted, owing to the monomer adaptability for reversible addition–fragmentation chain transfer polymerization. This method may simplify the fabrication procedures for many other polymeric FONs. Notably, the size, morphology, and fluorescence properties of these FONs are difficult to be controlled, which should be a direction of future efforts.

Carbonaceous dots represent superior fluorescent nanomaterials owing to their intrinsic advantages, including excellent luminescence property, photostability, water solubility, and good biocompatibility.^44^ Gao's group reported blue‐emitting fluorescent PDA dots (PDs) by means of hydroxyl radical‐induced reduction of π–π stacking interaction in PDA NPs.^45^ The characterization assay indicated that the resultant PDs were composed of 5,6‐dihydroxyindole, dopamine, and trihydroxyindole units, exhibiting broad adsorption and excitation‐dependent emission behavior, with desirable quantum yield (1.2%) and a maximal fluorescence at 440 nm. However, the luminescence excitation and emission wavelengths of this nanoprobe are still relatively short, which confines their applicability in bioimaging. To further improve the fluorescence efficacy with a red‐shift wavelength, this group described a one‐step hydrothermal method for fabricating melanin‐originated carbonaceous dots (MCDs).^46^ The as‐prepared MCDs showed broad absorption around 200–400 and 500–700 nm, and exhibited strong red shift emission at 570 and 645 nm, respectively. Fluorescence imaging of C6 cells revealed the great potential of MCDs in bioimaging applications. Subsequently, the aforementioned MCDs were used for high‐performance tumor fluorescence imaging in vivo, showing efficient accumulation within the 4T1 tumor region.^47^

#### 4.1.2 Photoacoustic Imaging (PAI)

PAI is an emerging noninvasive imaging modality in the field of disease diagnosis. It offers higher spatial resolution and sensitivity and increased tissue penetration depth compared with traditional optical techniques. As the generated thermal energy is related to the light energy absorbed by specific molecules, the photoacoustic signal strength is determined by the optical absorption at specific wavelengths. Since MelNPs possess intense broadband optical absorption extending to the NIR region, they are well suitable as exogenous photoacoustic contrast agents. For instance, Liopo et al. described polyethylene glycol (PEG) modified melanin‐like nanoparticles (MNP) for optoacoustic tomography contrast enhancement.^48^ The tubing phantoms exhibited satisfactory imaging resolution (<300 μm) and sensitivity (∆μ_a_ = 0.03 cm^−1^) upon a low laser energy density at 20 mJ cm^−2^. Under equal optical absorption condition, their optoacoustic efficiency was comparable to that of traditional gold nanorods. Given that the chemically inert and dense network structure of natural melanins restrict their enzymatical degradation, a controlled covalent form of PDA was obtained via facile Kumada‐coupling approach, which could be oxidized into line and water‐soluble melanin.^49^ High photoacoustic contrast was achieved with a detection depth beyond 10 mm. Considering the superior biodegradability and less biotoxicity, they hold great potential for PAI applications.

Despite the potential of MelNPs as a photoacoustic nanoprobe, the monotonic near‐infrared (NIR) absorption decrease leads to limited imaging sensitivity. To overcome this limitation, Ju et al. introduced hydrolysis‐susceptible citraconic amide onto the MelNPs surface.^50^ The resultant pH‐MelNPs could aggregate spontaneously with one another under mild acidic condition, resulting in an remarkably increased photoacoustic signal intensity (8.1 times higher) than that in neutral environment at 700 nm without tuning the absorption. This aggregation‐induced photoacoustic signal amplification can be explained by the overlapping thermal fields of the pH‐MelNPs. This stimuli‐sensitive photoacoustic signal amplification strategy could be expanded to tumor specific imaging in response to the acidic tumor microenvironment. Recently, Longo et al.^51^ reported novel melanin derivatives with high water solubility as photoacoustic contrast agents. The melanin free‐acid were obtained via light oxidative breakdown synthetic melanin granules, without requiring sonication steps in traditional synthetic strategies. Benefitting from their small sizes, PEG‐functionalized melanin free‐acid demonstrated satisfactory results for assessing tumor microvasculature following antiangiogenic treatment using dynamic contrast enhanced PAI.

**Figure 3:**
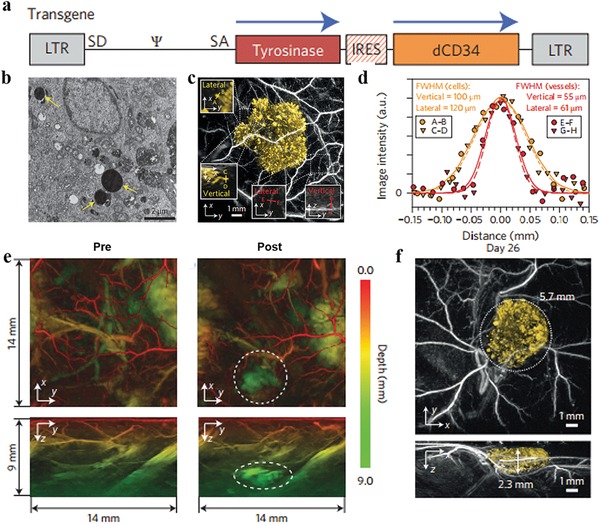
Deep in vivo PAI of xenograft tissues using a tyrosinase‐based reporter system. a) Schematic diagram of SFG‐based retroviral vector. b) TEM images of pigmented cytoplasmic granules in Tyr‐expressing 293T cells. Scale bar, 2 µm. c) In vivo horizontal (*x*–*y*) MIP images of Tyr‐expressing K562 cells and surrounding vasculatures acquired immediately post subcutaneous injection (λ_ex_ = 600 nm). d) Spatial resolution analysis by lateral and vertical profiles through the vessels in (c). e) *x*–*y* and *z*–*y* MIP images of the mouse hind leg and abdomen prior to and post injection of Tyr‐expressing K562 cells (λ_ex_ = 680 nm). f) *x*–*y* and *y*–*z* MIP images of Tyr‐expressing 293T cells obtained on day 26 post subcutaneous inoculation. Abbreviations: Tyr, tyrosinase; MIP, maximum intensity projection. Reproduced with permission.^52^ Copyright 2015, Springer Nature.

Despite appreciable success, synthetic MelNPs often suffer from relatively low solubility and high cost for pilot scale production. As tyrosinase catalyzes the biosynthesis of eumelanin from cellular tyrosine, the transgenic expression of Tyr providing genetically encoded photoacoustic contrast holds great promise. It possesses several advantages including robust stability in circulatory system, customization by specific genetic modification, and easy production in large‐scale. For instance, high contrast and stable transduction were achieved by using retroviral vector co‐expressing Tyr and truncated human CD34. For the first time, the longitudinal photoacoustic visualization of the cell population growth of genetically labeled 293T cells in vivo over a period of 26 days was achieved, with long imaging depth (near 10 mm) and high spatial resolution (<100 µm, **Figure** **3**).^52^ Owing to the elevated expression levels of Tyr and intense optical absorption of eumelanin, the Tyr‐expressing cells were unambiguously distinguished from surrounding microvasculature at an excitation wavelength of 640 nm. The excellent image contrast and imaging depth provide new opportunities for spatial–temporal monitoring of cell growth dynamics and complicated biological processes. In another study, biopolymer–melanin encapsulated bacterial outer membrane vesicles (OMV^Mel^) were produced for PAI applications, by using an *Escherichia coli* strain overexpressing a Tyr transgene.^53^ The as‐obtained the vesicles exhibited strong optoacoustic signals in phantoms. After systemic administration, OMV^Mel^ could accumulate within the tumor region in a passive targeting manner. Using multispectral optoacoustic tomography, the tumor‐associated OMV^Mel^ spatiotemporal distribution was noninvasively monitored in vivo. The bioengineered vesicles are promising to act as potent alternatives to commonly chemosynthetic nanomaterials for PAI, and this biological design strategy can be extended to many other types of genetically encoded agents.

#### 4.1.3 Magnetic Resonance Imaging (MRI)

MRI represents one of the most prominent and prevailing imaging modalities in clinic. Although MRI owns splendid soft tissue contrast, high temporospatial resolution, and unlimited penetration depth, it encounters relatively low sensitivity. Paramagnetic metal ion‐based MR contrast agents can remarkably improve the imaging sensitivity and diagnostic accuracy via accelerating the longitudinal or transverse relaxation time of proximal water protons. On account of the inherent metal ions chelating property of melanin, melanotic melanomas showed significant hyperintense on *T*_1_‐weighted MR images.^54^ As shown previously, Gd^3+^ chelated melanin, extracted from fully fermented tea leaves (*Thea sinensis Linn*.), could serve as oral MR contrast agents for gastrointestinal enhancement.^55^ Inspired by this, MelNPs have been extensively employed as building blocks for chelating various paramagnetic metal ions as MR contrast agents, without requiring additional extrinsic chelators. This is attributed to the abundant functional groups including amine, carboxy, *o*‐quinone, semiquinone, and phenolic groups.^13^ Examples of metal ions incorporated MelNPs as MR contrast agents are shown in **Table** **1**.

**Table 1:** Examples of metal ions incorporated MelNPs as MR contrast agents

Reference	MelNPs	Metal ions	Metal loading [wt/wt]	Magnetic field	*r*_1_ [mm ^−1^ s^−1^]	*r*_2_ [mm ^−1^ s^−1^]
[qv: 36b]	Gd‐DTPA‐Dpa‐melanin CNSs	Gd^3+^		1.5 T	6.9	
[qv: 28b]	PEG‐Fe^3+^‐MelNPs	Fe^3+^	0.72%	3.0 T	17	18
[qv: 28d]	^64^Cu‐Fe‐RGD‐PEG‐MNP	Fe^3+^	90 Fe^3+^ ions per MNP	1.0 T	1.2	
[qv: 57a]	M_G_L_R_‐MNP(Fe)‐ HER2	Fe^3+^		0.47 T	6.7	
[qv: 28c]	h‐Au‐melanin‐PEG‐Fe^3+^	Fe^3+^		4.7 T	7.8	10.3
[qv: 57b]	PEG‐Fe‐PDA NPs	Fe^3+^	1.12%	1.5 T	5.4	
56	PMPDA NPs	Mn^2+^		9.4 T	6.55	
26	TRITC‐Gd‐Mel@SiO_2_ NPs	Gd^3+^	5.4%	7.0 T	14.3	
[qv: 57c]	PDA‐Fe^3+^‐ICG NPs	Fe^3+^	0.83%	3.0 T	14	83.3
27	PDA‐ICG‐PEG‐DOX(Mn)	Mn^2+^		3.0 T	14.15	39.2
73	^64^Cu‐MMNs	Fe		7.0 T		167.28
61	MNP‐Gd^3+^	Gd^3+^		3.0 T	1.9684	
[qv: 28a]	PDAs@CP_3_‐DOX	Fe^3+^	6.76%	1.5 T	7.524	45.92
[qv: 58b]	MNP‐PEG‐Mn	Mn^2+^		3.0 T	20.56	
[qv: 28e]	iMNP	Fe^3+^		0.47 T	6.2	
64	CDPGM	Gd^3+^		7.0 T	14.06	
[qv: 58a]	MNP‐Mn	Mn^2+^	59 Mn^2+^ ions per MNP	3.0 T	18.86	
154	AlgPDA(Ca/Mn) NG	Mn^2+^	0.16%	7.0 T	12.54	
155	Lip‐Mel	–		7.0 T	0.25	
156	PDA/Fe‐ALN	Fe^3+^	5.3%	7.0 T	5.74	
157	euMel‐Fe_3_O_4_ NPs	Fe		7.0 T		245.88
[qv: 36a]	PMnEMNPs	Mn^2+^	10.2%	1.5 T	60.8	52.2
				3.0 T	36.8	82.1
				7.0 T	14.2	145.4
65	MMPP	Mn^2+^	0.88%	4.7 T	46.5	
158	MnCO@MPDA NPs	Mn^2+^		3.0 T	10.47	

Usually, these MelNPs were first fabricated via enzymatic or chemical oxidation and polymerization of applicable precursors (e.g., tyrosine, dopamine) in alkaline environment (e.g., ammonium hydroxide, sodium hydroxide) and specific organic solvents.[qv: 28a,36b,56] Then, metal ions (e.g., gadolinium,^26^ manganese,^27^ and iron[qv: 28b]) were anchored onto the as‐prepared MelNPs. This procedure is designated as post‐polymerization doping strategy. For instance, Ju et al. reported bio‐inspired Fe^3+^‐MelNPs with high *r*_1_ relaxivity (17 mm ^−1^ s^−1^, 3.0 T).[qv: 28b] Subsequently, Fe^3+^ ions chelated MelNPs have been widely explored for *T*_1_‐weighted tumor imaging.[qv: 28c,e,57] These nanoprobes demonstrated significant MR contrast enhancement, confirmed by both in vitro and in vivo experiments. Considering that Mn^2+^ ions are essential elements for the metabolism of organisms, Mn^2+^‐chelated MelNPs have also received much attention as MR contrast agents.^27^, ^56^, ^58^ Likewise, Cu^2+^‐loaded PDA NPs could act as an effective *T*_1_ contrast agent for environment‐responsive tumor imaging at 1.5 T (5.39 and 3.66 mm ^−1^ s^−1^ at pH 7.4 and pH 6.5, respectively).^59^ This was attributed to the increased electron–proton dipole–dipole interaction correlation time and the Cu^2+^ release in acidic environment. Given that ultrasmall‐sized nanoparticles own deeper tumor tissue penetration and can be easily metabolized from in vivo, Mn^2+^‐chelated ultrasmall melanin nanoparticles (MNP‐PEG‐Mn) with desirable water solubility and high *r*_1_ relaxivity (20.56 mm ^−1^ s^−1^ at 3.0 T) were reported recently.^60^ The relaxivity was much higher compared with the existing manganese based *T*_1_ MR contrast agents. As expected, MNP‐PEG‐Mn showed wonderful tumor‐targeted imaging and rapidly efficient clearance via hepatobiliary and renal pathways, due to their ultrasmall size (diameter 5.6 nm). Subsequently, this group presented Gd^3+^‐chelated ultrasmall (diameter 7 nm) water‐soluble melanin nanoparticles (MNP‐Gd^3+^) for MR monitoring and tracking of stem cells in vivo.^61^ The MNP‐Gd^3+^ showed high cell labeling efficiency toward bone mesenchymal stem cells by means of endocytosis. The MNP‐Gd^3+^‐labeled stem cells could be even detected after four weeks post intramuscular injection using 3.0 T MRI.

**Figure 4:**
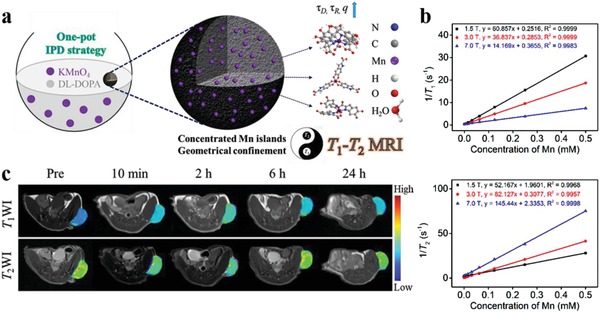
Manganese doped eumelanin‐like coordination nanocomposites for *T*_1_ *–T*_2_ dual‐modal MRI. a) Schematic diagram of the synthesis procedure and contrast enhancement mechanisms of MnEMNPs. b) *r*_1_ (upper) and *r*_2_ (lower) relaxivity of MnEMNPs aqueous solution at different magnetic fields. c) In vivo *T*_1_WI and *T*_2_WI of U87MG tumor as a function of time post intravenous injection of PEGylated MnEMNPs. Reproduced with permission.[qv: 36a] Copyright 2018, Wiley‐VCH.

Despite promising, the post‐polymerization doping strategy suffers from relatively complicated chelation and purification processes, dissatisfactory metal loading efficiency (<1%), and metal ion detachment risk from the surface of MelNPs. Rationally engineering water soluble MelNPs with high relaxivity via simple procedures is of high interest while remains challenging. In view of this, Liu et al. utilized a facile one‐pot intrapolymerization doping strategy to develop novel water‐soluble manganese doped eumelanin‐like nanocomposites (MnEMNPs) in aqueous phase (**Figure** **4**).[qv: 36a] It was realized through straightforward chemical oxidation–polymerization of 3,4‐dihydroxy‐dl‐phenylalanine with potassium permanganate acting as the manganese source and oxidant simultaneously, without requiring extrinsic chelators or additional chelation processes. The as‐obtained MnEMNPs possessed ultrahigh *r*_1_ and *r*_2_ relaxivity, arising from the high manganese loading efficiency (10.2%) and geometrically confined conformation. The *r*_1_ relaxivity of MnEMNPs were 60.8, 36.8, and 14.2 mm ^−1^ s^−1^ at 1.5, 3.0, and 7.0 T, respectively, much higher than that of clinical gadolinium‐based contrast agents (gadopentetate dimeglumine, 6.78, 6.28, and 3.79 mm ^−1^ s^−1^ at 1.5, 3.0, and 7.0 T, respectively) and manganese ion standards (9.88, 8.59, and 4.88 mm ^−1^ s^−1^ at 1.5, 3.0, and 7.0 T, respectively). The relaxivity variations of MnEMNPs at various magnetic fields (from 1.5 to 9.4 T) resulted in increased *r*_2_/*r*_1_ ratios (from 0.86 to 10.79). As expected, PEGylated MnEMNPs showed satisfactory imaging performance for *T*_1_ *–T*_2_ dual‐modal contrast in U87MG glioma bearing mice. Compared with single *T*_1_ or *T*_2_ imaging, this dual‐modal contrast can afford synergistic and complementary image information, granting self‐confirmed and false‐free superiority. This facile, convenient, and efficient intrapolymerization doping approach would offer opportunities for developing novel high‐performance diagnostic nanoprobes in biomedicine.

#### 4.1.4 Nuclear Medical Imaging

Nuclear medical imaging, mainly refer to positron emission tomography (PET) and single‐photon emission computed tomography (SPECT) imaging, possesses high imaging sensitivity and easy quantification, and has been widely applied in clinical practice. The abundant functional groups on MelNPs render them as convenient and ideal building blocks for anchoring with various radionuclides (e.g., ^99m^Tc,^62^ ^131^I,^62^, ^63^ ^64^Cu^2+^,[qv: 28d,64] and ^89^Zr^65^) via different methods. This enables innovative design of MelNPs‐based nanoplatforms to realize radionuclide imaging and radiotherapy (RT).

For instance, ^89^Zr (half‐life: 78.4 h) was chelated onto ultrasmall Mn^2+^‐chelated melanin nanoparticles (^89^Zr‐MMPP) without requiring extrinsic chelators.^65^ The labeling yield was determined to be 87.9 ± 1.2% within 15 min incubation, and the ^89^Zr‐MMPP exhibited high stability in physiological solutions. After intravenous injection, PET imaging signals and quantitative analysis showed preferential renal accumulation of ^89^Zr‐MMPP in acute kidney injury mice than the healthy mice group. This was helpful to explain the antioxidative protection of MMPP against acute kidney injury. Similarly, inspired by the inherent metal ions chelation capacity of melanin, radioactive ^64^Cu^2+^ could be chelated onto MelNPs via simple mixing for radionuclide imaging, biodistribution monitoring, guiding therapy, and therapy response assessment.[qv: 28d,64]

In a study by Zhong et al., radionuclide ^99m^Tc (half‐life: 6.02 h) was chelated onto PDA‐PEG NPs (^99m^Tc‐PDA‐PEG) by simple magnetic stirring of ^99m^TcO_4_^−^ and PDA‐PEG mixed solution in the presence of stannous chloride.^62^ Furthermore, ^131^I (half‐life: 8 d) was chelated onto PDA‐PEG NPs (^131^I‐PDA‐PEG) using chloramine‐T oxidation labeling method. The labeling yield of ^99m^Tc and ^131^I were about 99% and 70%, respectively, and they both exhibited excellent radio‐stability in physiological solutions. After intravenous injection into 4T1 tumor‐bearing mice, SPECT imaging and gamma counter quantitative analysis revealed effective tumor accumulation of ^99m^Tc‐PDA‐PEG. The blood circulation and biodistribution of ^131^I‐PDA‐PEG were monitored using gamma counter. The results indicated long blood circulation time and substantial tumor uptake of ^131^I‐PDA‐PEG. Similarly, Li et al. also reported ^131^I labeled PDA NPs via electrophilic substitution reaction for gamma imaging and radiotherapy.[qv: 63a]

Recently, Yang's group reported ^131^I‐labeled melanin nanoparticles (MNP‐Ag‐^131^I) via a simple Ag‐I two‐step labeling approach.^66^ In this strategy, sliver ions were first chelated onto MNPs, and then ^131^I ions were added to react with sliver ions to form AgI. Compared with previously reported chloramine‐T oxidation labeling method, the as‐developed Ag‐I method exhibits superiorities including shortened labeling time, improved labeling yield and stability. As expected, the as‐obtained MNP‐Ag‐^131^I demonstrated excellent imaging capacities for both SPECT and Cherenkov radiation imaging. At 8 h post intratumoral injection into PC3 tumor‐bearing mice, Cherenkov imaging results showed that the total radiance intensity in the MNP‐Ag‐^131^I group was 20 times stronger than the ^131^I group, indicating much prolonged retention time for the former. Taken together, the radiolabeling and molecular loading capacities of PDA NPs offer opportunities to construct multifunctional nanoplatforms for improved theranostics.

#### 4.1.5 Multi‐Modal Imaging

Different imaging modalities have their own merits and drawbacks. With the rapid development of advanced biomedical imaging techniques, multi‐modal imaging has aroused intensive interest in the field of biomedical imaging. It can provide complementary diagnostic information through integrating the synergistic advantages of different imaging modalities.^67^ Unfortunately, conventional multi‐modal nanoprobes require complicated and time‐consuming synthetic procedures to integrate different imaging contrast components onto a single nanoplatform. In addition, toxic reagents are usually introduced and raise potential safety issues, which hinder their further application and clinical setting. In view of this, developing easily prepared nanoplatforms with intrinsically multi‐modal imaging properties and desirable biosafety is highly attractive.

**Figure 5:**
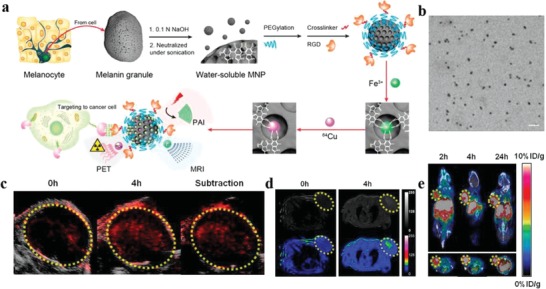
Ultrasmall melanin nanoparticles as a naturally active nanoplatform for tumor multi‐modal imaging. a) Schematic diagram of the synthesis procedure of ^64^Cu‐Fe‐RGD‐PEG‐MNPs and their applications for multi‐modal imaging. b) TEM images of PEG‐MNPs. Scale bar, 20 nm. c) PA, d) MR, and e) PET‐CT images of U87MG tumor as a function of time post intravenous injection of ^64^Cu‐Fe‐RGD‐PEG‐MNPs (200 µL, 10 × 10^−6^ m. Reproduced with permission.[qv: 28d] Copyright 2014, American Chemical Society.

Benefitting from their native appealing properties, melanin holds great potential in providing insight into the development of multi‐modal imaging probes. The Raman spectra of natural melanin exhibit two characteristic intense signal peaks at around 1580 and 1380 cm^−1^, which was structurally similar to graphite.^68^ Inspired by this unique spectroscopic property, Ju et al. demonstrated that synthetic melanin (MR‐NP, M, and R indicates MRI and Raman imaging, respectively) could serve as MR and Raman dual‐mode contrast agents.[qv: 28c] Subsequently, this group developed novel hybrid multifunctional nanocomposites (M_G_L_R_‐MNP(Fe)‐HER2) consisting of microbubble, liposome, Fe^3+^‐chelated MelNPs, and antibody.[qv: 57a] The as‐prepared nanocomposites demonstrated satisfactory results for tumor ultrasound/MRI dual‐modal imaging. Owing to the intrinsic chelating capacity of melanin, different metal ions can be co‐loaded onto MelNPs for multi‐modal imaging. Based on ultrasmall‐sized (<10 nm) water‐dispersed MNPs, Fan et al. reported a cyclic c(RGDfC) peptide conjugated, and ^64^Cu^2+^, Fe^3+^ chelated multi‐modal biopolymer nanoprobe (**Figure** **5**).[qv: 28d] The as‐obtained MNPs showed high loading capacity and stability, with about 100 ^64^Cu^2+^, 90 Fe^3+^, and 8 c(RGDfC) molecules attached onto per nanoprobe, respectively. The ^64^Cu‐Fe‐RGD‐PEG‐MNPs showed desirable tumor accumulation via active targeting effect, and then could act as a triple‐modal nanoprobe for PAI/MRI/PET imaging in U87MG tumor‐bearing mice. Similarly, Ha et al. prepared bio‐inspired ion‐doped melanin nanoparticles (iMNPs), which were simultaneously doped with Bi^3+^, Fe^3+^, and I^+^.[qv: 28e] After functionalization with an epidermal growth factor receptor (EGFR) antibody, iMNPs‐EGFR significantly improved the performance of MRI/CT/SPECT triple‐modal imaging of EGFR‐overexpressed orthotopic HepG2 hepatocellular carcinoma in a mouse model.

In addition to MRI based multi‐modal imaging, the integration of deep penetration depth from PAI and high spatial resolution from Raman imaging onto one single platform is promising in imaging‐guided surgeries, which remains challenging.^69^ To this end, Lin et al. proposed a one‐pot doping method to design polypyrrole‐PDA (PPy‐PDA) hybrid as Raman and PA dual‐modal contrast agents.^70^ In this design, PDA was adopted as a dopant due to the advantages including tunable bandgap energy for manipulating optical properties, and broad‐band optical absorption induced “super quenching effect.” Because polypyrrole and PDA are produced with different polymerization mechanisms, they formed a hybrid through π–π stacking interactions by infusion of these two macromolecules at the nanoscale. Compared with polypyrrole, this bandgap engineering strategy enhanced the photoacoustic and Raman scattering signal amplitude of SiO_2_‐CS@PPy‐PDA hybrid by 2.4 and 3.2 times, respectively. The feasibility of this nanoprobe for PAI and Raman imaging was verified in Hela cells and A549 tumor‐bearing mice. Such a dual‐modal signal amplification effect was attributed to reduced optical bandgap energy through photoinduced intermolecular energy transfer process. However, the macroscopical mixture of polypyrrole nanoparticles and PDA NPs resulted in no enhancement effect on these two imaging signal intensities. With better understanding of the chain packing and molecular structures in semiconducting polymers, the general applicability of this bandgap engineering strategy can be extended to develop a series of semiconducting conjugated polymers based multi‐modal contrast agents.

### 4.2 PTT

PTT has increasingly represented a minimally invasive phototherapy paradigm since its emergence, featuring superiorities including higher controllability, specificity, efficiency, and less side effects compared with traditional surgery and chemotherapy approaches.^71^ To date, numerous nanosized photothermal conversion agents (PTCAs) featuring strong NIR absorption have been exploited for tumor ablation.^72^ Despite exciting progress, few of currently available PTCAs has achieved clinical implementation, resulting from their poor water dispersibility, difficult surface functionalization, low photothermal conversion efficiency, dissatisfactory cost effectiveness, and long‐term biosafety concerns. The intrinsically intense NIR (650–900 nm) absorption of melanins, in combination with their excellent biocompatibility and biodegradability, make them meet the criteria as potent PTCAs candidates to address these hurdles. Examples of MelNPs for imaging guided tumor PTT are shown in **Table** **2**.

**Table 2:** Examples of MelNPs for imaging guided tumor PTT

Reference	MelNPs	Synthetic method	Tumor model	Route of administration	Application
159	Pol‐Mel	Sonication on ice of Poloxamer407 and melanin mixture	CT26	Intratumoral injection	PTT
18	liposome‐BSM NPs	Extraction from black sesame seeds (*Sesamum indicum* L.)	Eca‐109	Intratumoral and subcutaneous injection	SLN mapping and PTT
[qv: 36b]	Dpa‐melanin CNSs	Oxidation and self‐polymerization of dopamine in a mixture containing water, ethanol, and ammonia	4T1	Intratumoral injection	MRI and PTT
56	PMPDA NPs	Oxidation and self‐polymerization of dopamine in a mixture containing water, ethanol, and ammonia	4T1	Hela cells in vitro	MRI and PTT
[qv: 57b]	PEG‐Fe‐PDA NPs	Reverse microemulsion method through the self‐polymerization reaction of dopamine	SW620	Intratumoral injection	MRI guided PTT
[qv: 38b]	Melanin@RBC	Extraction from cuttlefish	A549	Intravenous injection	PAI guided PTT
17	Melanin@RBC‐M	Extraction from cuttlefish	MCF‐7	Intravenous injection	PAI guided PTT
53	OMV^Mel^	Escherichia coli strain overexpressing a tyrosinase transgene	4T1	Intravenous injection	PAI guided PTT
[qv: 57c]	PDA‐Fe^3+^‐ICG NPs	NaOH neutralization of dopamine hydrochloride	4T1	Intravenous injection	MRI/PAI guided PTT
157	euMel‐Fe_3_O_4_ NPs	One‐pot co‐precipitation method	U87MG	Intratumoral injection	MRI/PAI guided PTT
155	Lip‐Mel	Self‐assembly of melanin and hybrid lipid	MDA‐MB‐231	Intravenous injection	MRI/PAI guided PTT
[qv: 58a]	MNP‐Mn	HCl neutralization of the melanin dissolved in NaOH	Hep‐2	Intratumoral injection	MRI/PAI guided PTT
[qv: 36a]	PMnEMNPs	Chemical oxidation–polymerization of the 3,4‐dihydroxy‐dl‐phenylalanine precursor with potassium permanganate	U87MG	Intravenous injection	*T*_1_–*T*_2_ MRI/PAI guided PTT
26	TRITC‐Gd‐Mel@SiO_2_ NPs	NaOH neutralization of dopamine hydrochloride	PC‐3	Catheter‐directed infusion	FLI/MRI guided PTT
73	^64^Cu‐MMNs	Biomimetic synthesis method using melanin to direct the co‐precipitation of Fe^3+^ and Fe^2+^ ions (molar ratio at 2:1) under alkaline condition	U87MG	Intravenous injection	PET/MRI/PAI/PTI guided PTT, UV and γ‐ray protection

Abbreviations: MRI, magnetic resonance imaging; PTT, photothermal therapy; PET, positron emission computed tomography; PAI, photoacoustic imaging; PTI, photothermal imaging; SLN, sentinel lymph node; FLI, fluorescence imaging.

For the first time, Liu et al. evaluated the feasibility of novel dopamine–melanin colloidal nanospheres (Dpa‐melanin CNSs) as PTCAs for tumor ablation.[qv: 36b] The as‐obtained Dpa‐melanin CNSs exhibited an excellent photothermal conversion efficiency up to 40%, which was much higher than that of many previously reported nano‐sized PTCAs. Following intratumoral injection into 4T1 tumor‐bearing mice and laser irradiation, Dpa–melanin CNSs showed significant tumor growth suppression effect. Inspired by the intrinsic photoacoustic property and excellent metal ion chelation ability, Mn^2+^‐chelated MelNPs have been widely explored for MRI/PAI guided PTT applications.[qv: 36a,56,58a] Similarly, Gd^3+^‐chelated and bioinert silica coated MelNPs (TRITC‐Gd‐Mel@SiO_2_ NPs) were also reported for MRI/fluorescence imaging guided tumor PTT.^26^ The silica nanocoating could not only provide improved *T*_1_ MR contrast comparing with bare Gd‐Mel NPs, but also prevent the innate fluorescent quenching effect of melanin when labeled with fluorescent molecules. Given the intense affinity of melanin to metal ions, radionuclide ^64^Cu‐coupled magnetic melanin nanoparticles (^64^Cu‐MMNs) were strategically fabricated by using commercialized melanin as the biotemplate.^73^ The resultant ^64^Cu‐MMNs demonstrated versatile theranostic capacities for PET/MR/PA multi‐modal imaging guided tumor thermal ablation, and ultraviolet and γ‐irradiation protection.

Compared with aforementioned synthetic biopolymers, natural melanin nanoparticles directly extracted from living organisms have received more alluring interest for biomedical applications. This is attributed to their native nontoxicity and easy preparation from abundant natural sources. For instance, sheet‐like structured black sesame melanin (BSM) molecules were obtained by extraction from natural black sesame seed skin (*Sesamum indicum L*.) for tumor PTT applications.^18^ After intratumoral administration and NIR laser irradiation, the liposome‐encapsulated BSM (liposome‐BSM) significantly suppressed Eca‐109 esophagus carcinoma growth. Interestingly, the subcutaneous injected liposome‐BSM nanocomposites demonstrated satisfactory results for sentinel lymph node mapping, identified by their recognizable staining effect. The nanocomposites own advantages over conventional small molecule organic dyes for determining the stage of tumor progression, owing to the long‐term entrapment in the sentinel lymph nodes and no phototoxicity for the former.

**Figure 6:**
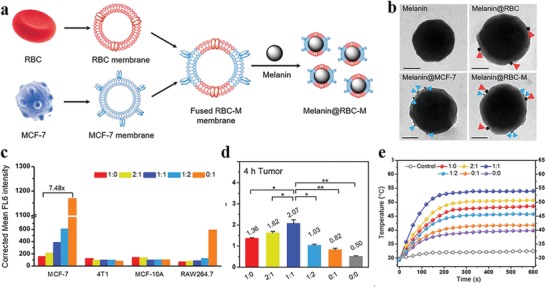
Erythrocyte‐cancer cell hybrid membrane‐shielded melanin nanoparticles for enhanced tumor PTT. a) Schematic illustration of the preparation procedure. b) Immunogold TEM images of various nanoprobes for CD47 (red) and CD340 (blue). Scale bars, 50 nm. c) Fluorescence intensity of various cells after treatment with DiD‐labeled Melanin@RBC‐M (20 µg mL^−1^, 4 h) with different RBC to MCF‐7 membrane protein weight ratios. d) Tumor accumulation profiles of Melanin@RBC‐M with different RBC to MCF‐7 membrane protein weight ratios at 4 h after intravenous injection (*n* = 3). *p <0.05, **p <0.01, ***p <0.001. e) Temperature variation profiles of MCF‐7 tumor during laser irradiation (808 nm, 1 W cm^−2^, 10 min) at 4 h after treatment with Melanin@RBC‐M with different RBC to MCF‐7 membrane protein weight ratios. Abbreviations: RBC, red blood cell. Reproduced with permission.^17^ Copyright 2018, Elsevier.

Despite enhanced permeability and retention (EPR) effect mediated tumor passive targeting, the majority of intravenously injected nanomaterials encounter easy recognization and rapid clearance by the reticuloendothelial system, leading to insufficient tumor accumulation and limited therapeutic performance.^74^ In this respect, erythrocyte membrane‐derived biomimetic coating has represented as a promising biomimetic strategy to enhance the penetration of nanomaterials across the biological barriers, due to the existence of “self‐markers” (e.g., CD47 protein, glycans, peptides, acidic sialyl) and the release of “do not eat me” signal.^75^ In a recent study, Yang and his co‐workers developed living cuttlefish originated and erythrocyte membrane‐camouflaged melanin (Melanin@RBC) nanoparticles as novel high‐performance PTCAs.[qv: 38b] RBC membrane coating maintained the excellent photothermal conversion property of pristine melanin nanoparticles. Compared with the latter, Melanin@RBC showed excellent photothermal capacity, prolonged blood circulation, promoted immune evasion and improved tumor accumulation in A549 tumor‐bearing mice. To further enhance the PTT efficacy, this group subsequently designed erythrocyte‐MCF‐7 cell hybrid membrane‐camouflaged melanin (Melanin@RBC‐M) nanoparticles, endowing the nanoparticles with prolonged blood circulation (from RBC membrane) and homologous tumor targeting ability (from MCF‐7 cell membrane) simultaneously (**Figure** **6**).^17^ The blood circulation profile and homotypic targeting ability of Melanin@RBC‐M could be well controlled by adjusting the membrane protein weight ratios of these two membrane components. The resultant Melanin@RBC‐M (RBC to MCF‐7 membrane protein weight ratio = 1:1) exhibited optimal tumor accumulation and superior PTT efficacy, benefiting from the optimal balance between homotypic targeting and prolonged blood circulation. Considering the favorable biocompatibility of both native melanin and cell hybrid membrane, this hybrid membrane camouflage strategy represents a feasible and effective approach to improve the controllability and flexibility of melanin nanoparticles for personalized medicine.

### 4.3 Drug Delivery for Tumor Treatment

The majority of small molecular therapeutic agents encounter poor water solubility, transient blood circulation time, and nonspecific biodistribution in vivo, inevitably leading to poor bioavailability and unfavorable therapeutic outcomes.^76^ Additionally, long‐term continuous administration throughout the treatment period usually induces multidrug resistance and severe systemic adverse reactions, leading to deteriorative condition and endless suffering to patients. With the rapid development of nanotechnology, custom‐designed drug nanocarriers have showed great potential for improved solubility and stability, maximal tolerated drug dosage, pharmacokinetics, and bioavailability of therapeutic compounds, eventually enhanced therapeutic efficacy. Most solid tumors possess significantly increased vascular permeability and suppressed lymphatic drainage compared to normal tissues. As a result, nanocarriers can passively accumulate and preferentially retain within the tumor region mediated by the well‐known EPR effect.^77^ And the nanocarriers can bypass the drug efflux pumps (e.g., P‐glycoprotein) to some extent, leading to elevated intracellular drug concentration. More interestingly, prolonged circulation half‐life and controllable drug release can be realized by using functionalized nanocarriers, which could further reduce the adverse reactions and medical consumption. Frequently used nanocarriers mainly include liposomes, micelles, dendrimers, organic polymer nanoparticles, inorganic mesoporous silica nanoparticles, and calcium‐based nanomaterials, and so forth.^78^ Unfortunately, they themselves are usually monofunctional and not chemically reactive, making the functional modification and in vivo real‐time tracking difficult. Moreover, concerns about dissatisfactory biodegradability, potential immunogenicity, and long‐term toxicity impede their further clinical implementation.

The exploration and development of MelNPs have substantially broadened new horizons in exploiting novel drug delivery systems. This is attributed to the following advantages of MelNPs: i) As previously described, melanins are natural nontoxic biopolymers with plentiful π‐conjugated structures on their surface (e.g., dihydroxyindole, indolequinone). This affords high payloads with various aromatic structured drugs via π–π stacking, hydrogen bonding interaction, and van der Waals interaction.^29^ This process is quite simple and does not involve any chemical intervention, while the loading efficiency largely depends on the surface of MelNPs. Moreover, the drugs can be conjugated onto the surface of MelNPs by chemical bonding,^30^ or encapsulated within the polymer matrix of MelNPs.^31^ This could protect the loaded drugs from enzymatic degradation in complex physiological environment. ii) On the other hand, MelNPs have abundant chemically active functional groups (e.g., catechol, amine, imine, quinone) on their surface, allowing secondary modification with a variety of thiol‐ and amino‐containing molecules by means of Michael addition, Schiff base reaction and coordinative interactions.^23^ PEGylation (e.g., —SH or —NH_2_ terminated PEG) and tumor‐specific targeting modules (e.g., hyaluronic acid, folate, arginine‐glycine‐aspartic acid (RGD) peptide) functionalization can give rise to additional special functions such as reduced reticuloendothelial system uptake, prolonged blood circulation and improved tumor accumulation, and thus realize enhanced therapeutic efficacy as well as reduced side effects.^79^ iii) Additionally, the in vivo biodistribution of MelNPs can be dynamically monitored by MRI and PAI apart from their usage as nanocarriers, achieving theranostics. More interestingly, the excellent photothermal conversion capability of MelNPs provides possibility for PTT‐incorporated combination therapy onto one nanoplatform. The PTT‐induced mild hyperthermia can accelerate tumor blood flow and intracellular drug release, which are beneficial to enhanced tumor oxygenation and cell‐killing effect, respectively. iv) The last but not least, the unique characteristics of tumor microenvironment can provide endogenous stimuli (e.g., acidic pH, elevated hydrogen peroxide and glutathione levels) for tumor‐specific drug delivery and controllable drug release, which can avoid premature drug leakage at non‐targeted sites and is crucial to minimize potential adverse effects. In view of this, these versatile functionalities make MelNPs as a novel, general and promising theranostic nanoplatform by combing accurate diagnosis and effective treatments of tumors. Examples of MelNPs as nanocarriers for drug delivery and controlled drug release are shown in **Table** **3**.

**Table 3:** Examples of MelNPs as nanocarriers for drug delivery and controlled drug release

Reference	MelNPs	Drug loaded	Loading capacity	Cell line in vitro	Application
[qv: 83c]	Lys_FITC_ ‐PGA_PDA15_ capsules	Lysozyme	–	–	Protease‐responsive drug release
[qv: 83d]	Dox‐loaded AF488‐labeled PDA capsules	DOX	–	HeLa cells	pH‐responsive drug release
160	CPT‐loaded PDA	CPT	1.46 µmol CPT on 1 mg PDA NPs	A549 and HeLa cells	pH‐responsive drug delivery
161	FA‐PEI‐PDA/DOX	DOX	92%	HeLa cells	Folate receptor targeted drug delivery
81	Melanin extracted from cuttlefish	MZ	2.3%	Caco‐2 cells	pH‐responsive drug release
82	DOX‐loaded mfp‐1 NPs	DOX	50–75%	HeLa cells	pH‐responsive drug delivery
162	PDMAEMA‐g‐PDA microcapsules	Av	52.7%	–	Temperature‐controlled release
163	PDA‐R	Rifampicin	76%	–	Nanoreservoir
122	Cipro‐loaded NP–gel hybrid	Cipro	–	–	localized antimicrobial drug delivery
164	PDA‐PEG‐TPP‐DOX	DOX	5%	MDA‐MB‐231 cells	Mitochondria‐targeted drug delivery
129	PDA‐TH/TOCNFs hydrogel	TH	14.4%	–	pH/NIR‐responsive and long period drug release
165	HA‐Cys/PDA‐DMOG hydrogel	DMOG	10%	HUVECs	Angiogenic drug delivery and sustained release
166	DOX‐PEG‐MNPs	DOX	35%	MDA‐MB‐231 cells	Controlled and prolonged release
123	Met encapsulated PDANPs	Met	45.2 ± 5%	SH‐SY5Y cells	Drug delivery
[qv: 63b]	^64^Cu labeled SRF‐MNPs	SRF	25%	HepG2	PET/PAI guided chemotherapy
84	HMP‐NPs	PTX	304.72 µg PTX in 800.48 µg melanin	MDA‐MB‐231	PAI guided chemotherapy

Abbreviations: DOX, doxorubicin; MZ, metronidazole; Av, avermectin; TH, tetracycline hydrochloride; Cipro, ciprofloxacin; DMOG, dimethyloxalylglycine; Met, metformin; CPT, camptothecin; SRF, sorafenib; PTX, paclitaxel.

#### 4.3.1 pH‐Responsive Drug Release

Effective and controlled release of drugs from nanocarriers at desired sites is a crucial precondition for achieving efficient therapeutic effect. In this respect, MelNPs represent an ideal drug nanocarrier candidate owing to the multi‐stimuli accelerated drug release behaviors, benefitting from the intrinsic characteristics of tumors and unique drug loading mechanisms of MelNPs.[qv: 14e] The release of drugs from MelNPs under normal condition is quite weak and slow owing to the strong interaction between MelNPs and loaded drugs, preventing unwanted drug leakage. After arriving at the tumor region, endogenous stimuli such as hydrogen peroxide and glutathione can disrupt the hydrogen bonding interaction and π–π stacking interaction between MelNPs and loaded drugs, respectively, accelerating the drug release. Moreover, NIR irradiation induced temperature increase of MelNPs could weaken the hydrogen bonding interaction between MelNPs and loaded drugs, triggering the drug release. Particularly, pH‐triggered drug release fashion has earned intense interest for controlled drug delivery applications. This is arisen from the fact that hypoxia, glycolysis, and excessive lactic acid levels produce a weakly acidic tumor environment (pH < 7.0), which is lower than the physiological pH in normal tissues (pH 7.2–7.4).^80^ MelNPs encounter additional environmental stimuli when endocytosis into more acidic endosomal and lysosomal compartments (pH 5.0–6.0). Amino groups on MelNPs can undergo protonation under acidic condition, which may disrupt the π–π stacking interaction between MelNPs and loaded drugs, leading to accelerated drug release. From the above, MelNPs are promising to serve as pH‐responsive drug transport vehicles for enhanced therapeutic efficacy as well as reduced systemic adverse effects.

**Figure 7:**
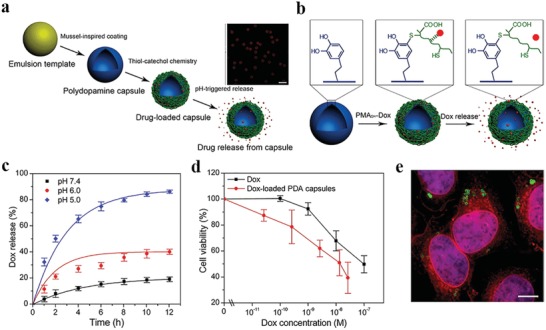
Immobilization and intracellular delivery of doxorubicin from PDA capsules. a) Schematic diagram of the synthesis procedure of PDA capsules and their pH‐responsive drug delivery applications. The insets show fluorescence images of AF488‐labeled DOX‐loaded PDA capsules. Red, DOX. b) Immobilization and pH‐responsive DOX release. c) Time‐dependent DOX release behavior from PDA capsules under various pH conditions. d) Relative cellular viabilities of Hela cells after receiving different treatments. e) Fluorescence images of HeLa cells after incubation with DOX‐loaded AF488‐labeled PDA capsules. Red, DOX; green, PDA capsules; blue, nuclei. Reproduced with permission.[qv: 83d] Copyright 2012, American Chemical Society.

For the first time, Araújo et al. described natural nanosized melanin extracted from cuttlefish ink sacs as a pH‐responsive drug carrier.^81^ The antibiotic drug metronidazole was straightforwardly impregnated onto MelNPs using clean supercritical carbon dioxide technology. The as‐prepared drug–melanin formulations exhibited a pH‐responsive drug release behavior, identified at physiologic (pH = 7.4) and acidic (pH = 2.2) conditions. The dominant mechanism was found to be non‐Fickian transport. Subsequently, Kim et al. synthesized DOX‐loaded Fe^III^‐DOPA NPs through a co‐electrospray process.^82^ The pH‐responsive drug release was achieved by pH‐responsive changes in the Fe^III^‐DOPA coordination stoichiometry. The DOX‐loaded polymeric NPs showed enhanced cytotoxicity on Hela cells following cellular internalization and intracellular release in vitro. Compared with PDA NPs, PDA‐based nanocapsules own larger specific surface area and inner space for loading guest molecules, which makes nanocapsules attractive for drug delivery. To this end, a series of PDA nanocapsules with tailored size and shell thickness have been successively developed by Caruso's group.^41^, ^83^ For instance, a maleimide hydrazone derivative of DOX was coupled to thiolated poly(methacrylic acid) via thiol‐maleimide chemistry, and then the pH‐cleavable polymer–drug conjugates were immobilized within PDA capsules via thiol‐catechol reaction (**Figure** **7**).[qv: 83d] The acid‐labile hydrazone bond was stable at physiological pH in extracellular space while readily degraded in acidic endosomal/lysosomal compartments. As expected, DOX loaded PDA capsules exhibited increased drug release with decreased pH values in vitro. After incubation in pH = 7.4 and pH = 5.0 solutions for 12 h, the cumulative DOX release were below 20% and over 85%, respectively. After internalization by Hela cells, they showed improved cancer cell‐killing efficacy compared with free DOX. This general strategy holds great promise for designing PDA capsules as tailored drug transport vehicles with pH responsivity.

#### 4.3.2 Nanocarriers for Monotherapy

##### Imaging‐Guided Chemotherapy

In clinic practice, only periodical and inaccurate observation is routinely performed for assessing tumor chemotherapy response, due to the lack of integration of imaging and therapy procedures. In this case, ineffective or excessive chemotherapeutic agents are often used, leading to unreasonable therapeutic regimens and unwanted side effects. By employing multifunctional nanoagents with theranostic ability, real‐time noninvasive monitoring of the pharmacokinetics of drugs and their localization at the target site can be realized. The delivery, release, and efficacy of drugs can be also monitored and evaluated noninvasively. This beneficial feedback is crucial for the optimization of personalized precision medicine and clinical translation. However, most of the conventional nanocarriers are monofunctional and lack contrast properties, and time‐consuming and complicated procedures are required to introduce exogenous contrast agents onto these nanoplatforms for imaging‐guided therapy.

As mentioned above, the intrinsic drug loading capacity, imaging capacity, and simple and mild preparation procedures of MelNPs make them ideal nanoplatforms for safe imaging‐guided chemotherapy. For instance, Cheng and co‐workers first reported the simple use of MNPs as a drug‐loading nanosystem for hydrophobic sorafenib (SRF) delivery.[qv: 63b] Owing to the native biocompatibility and biodegradability, inherited photoacoustic property, chelating ability to radioactive ^64^Cu^2+^ ions, and easy binding to aromatic drugs, PEG‐functionalized ^64^Cu^2+^‐labeled SRF‐MNPs with high loading capacity (0.25 mg SRF in 1.0 mg MNPs) were fabricated. The water‐soluble SRF‐MNPs showed a linear and gradual SRF release profile regardless of pH changes, according with the inherent character of π–π interaction. The as‐prepared nanocomplexes could be effectively delivered to the tumor site following intravenous injection, confirmed by PET/PAI dual‐modal imaging. As expected, SRF‐MNPs demonstrated high performance for PET/PAI guided chemotherapy in HepG2 hepatocellular carcinoma‐bearing mice. Attributed to the slow degradation of melanin in vivo and gradual SRF release from SRF‐MNPs, a dosage as low as 4 mg kg^−1^ via intravenous injection was sufficient to suppress the tumor growth. The effect was much higher than that of traditional oral administration of 20 mg kg^−1^ SRF. It was not difficult to found that the SRF‐MNPs could significantly decrease the therapeutic dosage and thus the side effects. Similarly, in another study, melanin and paclitaxel were simultaneously loaded on albumin nanoparticles (HMP‐NPs) through ethanol precipitation method.^84^ The as‐prepared HMP‐NPs showed high loading capacity, and high tumor accumulation, and thus showed satisfactory results for PAI and chemotherapy against MDA‐MB‐231 breast cancer, confirmed by both in vitro and in vivo experiments. All these evidences indicate MelNPs are appropriate to behave as a natural and convenient nanoplatform for imaging‐guided chemotherapy and potential clinical translation.

##### Hypoxia‐Resistant Photodynamic Therapy (PDT)

PDT, well‐regarded as a powerful tumor therapeutic modality in clinic, possessing advantages including noninvasiveness, controllability, and minimal side effects.^85^ As one of the most malignant phenotypes of tumor, extreme hypoxia often leads to type II PDT resistance in which oxygen is involved during the cell killing process.^86^ To address this issue, tumor‐targeted oxygen‐carrying and in situ oxygen‐generating strategies have been enthusiastically proposed aiming at improving the tumor oxygenation. Despite promising, limited oxygen loading efficiency, unsatisfactory targeting capacity, as well as safety concerns hamper their further applications.

Inspired by the natural oxygen‐carrying capacity of RBCs, Liu et al. designed biomimetic aggressive man‐made pseudo‐erythrocytes (AmmRBC) for self‐oxygen‐supplied PDT against hypoxia‐resistant tumor.^87^ For this purpose, biovesicles composed of RBC membrane were prepared by biomembrane recombination technology. Then, methylene blue‐carried hemoglobin–PDA complexes were skillfully encapsulated inside the biovesicles by using porous polycarbonate filters. The mean content of corpuscular hemoglobin in AmmRBCs was about ten times higher than that in natural RBCs. The hemoglobin–PDA complexes were easily accessible attributed to the noncovalent bonding. Owing to the strong antioxidation potency of polyphenol groups, the introduced PDA served as the antioxidative enzymes to effectively counteract the oxidation damage of oxygen‐carrying hemoglobin during circulation. Moreover, the strong binding capacity of PDA toward aromatic compounds was utilized to load methylene blue. In this design, AmmRBCs could act as a self‐oxygen‐supplied nanoplatform to overcome hypoxia‐associated PDT resistance. AmmRBCs retained favorable interfacial characters and immune clearance escape capacity of parent RBCs due to their identical membrane origin. AmmRBCs demonstrated effective tumor accumulation, sufficient in situ oxygen supply, and satisfactory PDT effect, resulting in complete tumor elimination without recurrence. By loading with different desired therapeutics, the as‐developed AmmRBCs are promising as a general duty oxygen‐self‐supplied nanoplatform which can be extended to overcome other hypoxia‐associated treatment resistance, such as chemotherapy and radiotherapy.

##### Antigen Delivery for Immunotherapy

Cancer vaccine is an effective approach to evoke immune responses in the field of immunotherapy. Unfortunately, most of unformulated antigenic peptides post subcutaneous administration rapidly enter the peripheral blood vessels and spread systemically, leading to insufficient antigen presentation and limited antitumor immunity. Biomimetic nanoparticulated vaccine formulations are increasingly attractive for remedying the vaccine restrictions, due to their more efficient antigen presentation capacity. Considering the immunoregulatory capacity of dopamine,^88^ MelNPs represent an ideal candidate to antigen delivery carriers for improved tumor immunotherapy. In a recent work, PDA NPs were used for subcutaneous antigen delivery and their anti‐tumor efficacy was evaluated.^89^ Ovalbumin was used as a tumor model antigen and was readily loaded on the surface of PDA nanoparticles (OVA@Pdop‐NPs), with a high loading efficiency of 754 µg mg^−1^. Compared with free ovalbumin, the resultant OVA@Pdop‐NPs dramatically enhanced cellular uptake of ovalbumin by bone‐marrow‐derived dendritic cells and exhibited lysosome escape phenomenon. After stimulation with OVA@Pdop‐NPs, the maturation of dendritic cells was promoted with significantly elevated expression levels of major histocompatibility complex, costimulatory molecules, and inflammatory cytokines. In the nanovaccine group, rapid and easy migration into the draining lymph nodes and prolonged residence of ovalbumin was achieved post subcutaneous injection in vivo. In ovalbumin‐MC38 colon tumor bearing mice, subcutaneously injected nanovaccine remarkably inhibited the tumor growth through activating ovalbumin‐specific CD8^+^ T cells mediated immune responses and alleviating the immunosuppressive tumor microenvironment. These results suggested the potential of MelNPs as a promising vaccine vehicle in tumor immunotherapy.

##### Microwave Thermal Therapy (MWTT)

Tumor MWTT is an emerging therapeutic modality, based on the absorption of electromagnetic fields at microwave frequencies, which can instantaneously and irreversibly destroy the tumor tissues through an extremely high and localized temperature increase (at least 60 °C).^90^ Moreover, the shape and dimension of the target site for thermal ablation can be foreseeable and well controlled by adjusting the microwave settings. Recently, MWTT has earned considerable interest owing to its noninvasiveness, deep penetration, side effect‐free character, and convenient manipulation. To improve the selectivity and targeting of MWTT, Tian et al. reported microwave susceptible ionic liquids loaded hollow PDA capsules (ILs/PDA) for enhanced tumor thermal ablation.^91^ The hollow PDA capsules were prepared by selective etching silica core of silica‐PDA core/shell nanoparticles, and then the ionic liquids 1‐butyl‐3‐methylimidazolium hexafluorophosphate were loaded through an ultrasonic method. The selected ionic liquids possessed high polarizability and unique ionic character, resulting in high sensitivity to microwave irradiation and excellent electromagnetic–thermal energy conversion efficiency. After intravenous injection of ILs/PDA nanocomposites in H22 tumor bearing mice, remarkable antitumor effect was achieved following one single microwave irradiation (1.8 W cm^−2^, 5 min). Histological analysis, blood routine, and serum biochemical test together indicated the excellent biocompatibility of as‐prepared ILs/PDA nanocomposites. Moreover, ILs/PDA nanocomposites exhibited a remarkable degradation behavior after incubation in simulated body fluids for one month, indicating their biodegradability. These appealing results reveal that PDA capsules hold great potential for loading other nanoscale microwave susceptible agents, aiming at enhancing the antitumor effect of MWTT.

#### 4.3.3 Bi‐Modal Synergistic Therapy

Current single tumor monotherapy approach (e.g., surgery, chemotherapy and radiotherapy) in clinic is unable to eliminate the tumor completely, primarily ascribed to the characters of tumors, including complexity, diversity, and heterogeneity. With better understanding of tumor biology and rapid developments of emerging therapeutic techniques, recent advancements in tumor therapy strategies have gradually shifted to multi‐modal combination therapy from traditional monotherapy. Cooperative enhancement interactions among multiple therapeutic approaches have been proven to provide new possibilities for remarkably superadditive (namely “1 + 1 > 2”) therapeutic performance, compared with respective individual treatments or their theoretical combination.^92^ Regarding bi‐modal synergistic therapy, two therapeutic modalities with different working mechanisms are integrated onto a single nanoplatform and mutually complementary for each other, achieving enhanced therapeutic output with reduced adverse effects. Benefitting from the multifunctionality of MelNPs, they have gained much popularity to serve as a combination therapy nanoplatform against refractory tumors. In this section, several types of MelNPs‐based bi‐modal synergistic therapy are presented, and corresponding synergistic work mechanisms are discussed. Examples of MelNPs as nanocarriers for tumor synergistic therapy are shown in **Table** **4**.

**Table 4:** Examples of MelNPs as nanocarriers for tumor synergistic therapy

Reference	MelNPs	Drug loaded	Loading capacity	Tumor model	Application
30	PDA‐Ce6 NPs	Ce6	–	HepG2	FLI guided PDT–PTT
100	PHPD‐NPs	Pheo‐a	–	MDA‐MB‐231	FLI guided PDT–PTT
31	PDA−Ce6 NPs	Ce6	14.2 ± 0.85 × 10^−6^ m Ce6 on 1 × 10^−9^ m PDA NPs	T24 cells	PDT–PTT
167	PPIF NPs	IR‐820	1.48%	HeLa cells	PDT–PTT
99	PATP NPs	T‐ZnPc	21%	4T1	FLI/MRI guided PDT–PTT
[qv: 97b]	PLDA‐DA NPs	DOX	66–73%	HepG2 cells	CT–PTT
95	PDA‐PEG/DOX, PDA‐PEG/SN38	DOX, SN38	33.0 for DOX and 10.6% for SN38	PC‐9	CT–PTT
96	Dox@PAH‐cit/PDA NPs	DOX	30%	PC3	FLI guided CT–PTT
168	PDA@CP‐PEG‐DOX	DOX	80%	MCF‐7	CT–PTT
169	OSA‐CS‐PHA‐DDP hydrogels	DDP	2.56%	4T1	CT–PTT
[qv: 98a]	PDA‐SN38/PEG hydrogel	SN38	11.8%	PC‐9	CT–PTT
156	PDA‐ALN/SN38	SN38	8.45%	MDA‐MB‐231‐Luc	CT–PTT
170	MPDA‐DOX@TPGS	DOX, TPGS	79% for DOX	MCF‐7/ADR cells	CT–PTT
171	PDA‐PEG‐CP	Cisplatin	20%	HeLa cells	CT–PTT
172	PDO+PD‐BTZ	BTZ	7%	CT26 cells	CT–PTT
27	PDA‐ICG‐PEG/DOX(Mn)	DOX	150% for DOX	4T1	MRI guided CT–PTT
59	CuPDA NPs	Cu^2+^	–	KB	MRI guided CT–PTT
[qv: 28a]	PDAs@CP3‐DOX	DOX	2.562 mmol DOX/g	Hela	*T*_1_/*T*_2_ MRI guided CT–PTT
[qv: 98c]	PDAC‐DOX conjugate	DOX	about 50%	MCF‐7	PAI guided CT–PTT
[qv: 98b]	DOX‐loaded PEG‐ICG‐PDA/mCaP H‐JNPs	DOX	73%	HepG‐2	PAI guided CT–PTT
[qv: 97a]	PDA‐RGDC/DOX	DOX	200% for DOX	HeLa	PAI guided CT–PTT
173	PDCNs	DOX	33%	HeLa	FLI/PAI guided CT–PTT
114	nano‐agent	Pt(II) metallacycle	20%	U87MG	FLI/PAI guided CT–PTT
64	CDPGM NPs	DOX	40%	U87MG	MRI/PAI/PET guided CT–PTT
174	BV/PTX@Au@PDA/DOX	PTX, DOX	90.6% for PTX and 13.7% for DOX	U14	CT–PTT, and triggered immune responses
108	pDA@aspirin@CM	aspirin	–	4T1	Anti‐inflammatory therapy–PTT
105	CuS@Melanin‐PEG/DOX	DOX	weight ratio of 1:1 for DOX and CuS	4T1	PAI guided CT‐RT
62	^131^I‐PDA‐PEG/DOX	DOX	66%	4T1	SPECT imaging guided CT‐RT
[qv: 63a]	^131^I‐PDA‐PEG‐SAN‐MET	SAN, MET	PDA‐PEG: SAN: MET weight ratio = 1:1:2	4T1	Radionuclide imaging guided CT‐RT
106	PDA‐PEG/Cur/Ce6	Cur, Ce6	30% for Ce6 and 15% for Cur	A549	PDT‐RT
109	IL‐PDA‐DOX	DOX	10.79%	H22	CT–MWTT
158	MnCO@MPDA NPs	MnCO	60.3%	HCT116	MRI/PAI guided CO therapy–PTT

Abbreviations: MRI, magnetic resonance imaging; CT, chemotherapy; RT, radiotherapy; PTT, photothermal therapy; PET, positron emission computed tomography; PAI, photoacoustic imaging; PTI, photothermal imaging; FLI, fluorescence imaging; MWTT, microwave thermal therapy; Ce6, Chlorin e6; Pheo‐a, pheophorbide‐a; T‐ZnPc, thymine modified zinc phthalocyanine; DOX, doxorubicin; SN38, 7‐ethyl‐10‐hydroxycamptothecin; DDP, cisplatin; TPGS, d‐α‐tocopheryl polyethylene glycol 1000 succinate; BTZ, bortezomib; PTX, paclitaxel; SAN, sanguinarine; MET, metformin; Cur, curcumin.

##### Chemotherapy–PTT

In traditional single chemotherapy, long‐term continuous administration of chemotherapeutics throughout the treatment period induces potential multi‐drug resistance and severe systemic adverse reactions, leading to deteriorative condition and endless suffering to patients.^93^ In another aspect, uneven hyperthermia distribution and intermittent NIR irradiation in single PTT could lead to incomplete ablation of the marginal tumor tissues. Recently, chemo‐photothermal combination therapy has represented one of the most prevalent strategies for tumor bi‐modality therapy. On account of that PTT is unable to eradicate distant metastatic tumor cells and chemotherapy is a systemic therapy paradigm, the combination of these two approaches is achievable against both local tumors and distant tumor metastasis. Aside from killing cancer cells directly via hyperthermia (>50 °C), a mild temperature rise (43–45 °C) arising from PTT was proven to efficiently improve the cell‐killing effect of chemotherapeutic agents. Possible mechanisms are mild hyperthermia‐mediated boosted cell membrane permeability, enhanced cellular uptake of drug‐loaded nanocarriers, accelerated intracellular drug release from nanocarriers, and strengthened drug sensitivity to tumor cells.^94^ This interpretation may bridge the interaction between chemotherapy and PTT for substantially improved antitumor effect.

**Figure 8:**
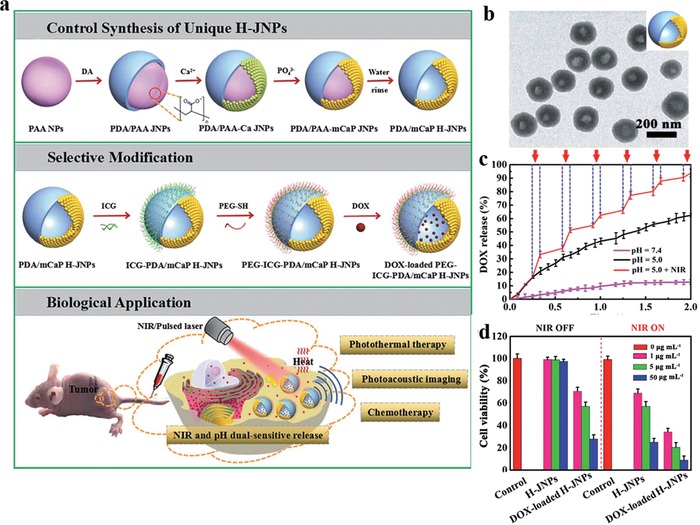
Multifunctional PDA mesoporous inorganic hollow Janus nanoparticles for PAI‐guided CT–PTT synergistic therapy. a) Schematic diagram showing the synthesis, of PEG‐ICG‐PDA/mCaP H‐JNPs and their theranostic applications. b) TEM images of PDA/mCaP H‐JNPs. Bar, 200 nm. c) pH and NIR laser (808 nm, 1 W cm^−2^) triggered DOX release profiles from the DOX‐loaded H‐JNPs. d) Relative cellular viabilities of HepG‐2 cells after receiving different treatments. Abbreviations: H‐JNPs, hollow Janus nanoparticles; PAA, poly (acrylic acid); mCaP, mesoporous calcium phosphate; DOX, doxorubicin. Reproduced with permission.[qv: 98b] Copyright 2017, Royal Society of Chemistry.

Encouraged by the excellent drug binding ability of melanin, DOX and 7‐ethyl‐10‐hydroxycamptothecin loaded PDA NPs have been reported for chemo‐photothermal synergistic therapy, showing a multiple stimuli‐responsive drug release behavior including NIR laser, acidic pH and reactive oxygen species (ROS).^95^ Likewise, controlled drug release was achieved by using pH‐responsive poly(allylamine)‐citraconic anhydride polymer.^96^ In another study, PDA NPs were developed as a versatile nanocarrier to afford simultaneous loading with multiple modules including indocyanine photosensitizer green, chemotherapeutic drug DOX and contrast agent manganese ions (PDA‐ICG‐PEG/DOX(Mn)).^27^ The as‐obtained nanoplatform demonstrated satisfactory performance for MRI guided chemo‐photothermal combination therapy in 4T1 tumor‐bearing mice. To further improve the tumor accumulation efficacy of nanocomposites, RGD peptide and lactose functionalized PDA NPs have been designed for tumor active targeting and chemo‐photothermal combination therapy.^97^ In addition to PDA NPs, hollow PDA capsules, PDA‐knotted poly(ethylene glycol) hydrogels, and PDA/mesoporous calcium phosphate hollow Janus nanoparticles (**Figure** **8**) have been innovatively developed as nanocarriers for PAI‐guided chemo‐phototherapy, exhibiting synergistic antitumor activity in vivo compared with the respective counterpart.^98^

Exploiting novel nanomaterials with excellent imaging capability is highly beneficial for precise guiding treatment. To overcome relatively low *r*_1_ relaxivity and Gd^3+^ ions detachment induced toxicity in gadolinium‐based MR contrast agents, radionuclide ^64^Cu labeled and DOX loaded PDA–gadolinium–metallofullerene core–satellite nanostructures (CDPGM NPs) were developed.^64^ In this design, NIR‐absorbing PDA core acts as PTCA, photoacoustic contrast agent, and a building block for assembling different functional modules simultaneously; gadolinium‐containing metallofullerene Gd_3_N@C_80_ plays as a satellite anchoring on the PDA surface for MRI; radionuclide ^64^Cu was chelated onto CDPGM NPs mediated by the strong affinity of catechol groups to copper ions for PET imaging. Finally, DOX was loaded as a model antitumor drug through hydrogen‐bonding and π–π stacking interaction. The as‐designed CDPGM NPs showed high *r*_1_ relaxivity (14.06 mm ^−1^ s^−1^, 7.0 T), low risk of Gd^3+^ ions detachment, and pH/NIR laser‐triggered drug release behavior. Effective CDPGM NPs accumulation at the tumor site was verified using MRI/PAI/PET multi‐modal imaging in vivo. Encouragingly, the tumor could be even completely eradicated without recurrence following chemo‐photothermal combination therapy. In another study, in order to obtain self‐validated diagnostic information, DOX loaded and coordination polymer encapsulated PDA nanocomplex (PDAs@CP_3_‐DOX) were developed for *T*_1_–*T*_2_ dual‐modal MRI‐guided chemo‐photothermal combination therapy.[qv: 28a] The Fe^3+^‐chelated PDA NPs could serve as *T*_1_ contrast agents (*r*_1_ = 7.524 mm ^−1^ s^−1^, 1.5 T), while the pure coordination polymer synthesized from Fe^3+^ and benzene‐1,3,5‐tricarboxylic acid could serve as *T*_2_ contrast agents (*r*_2_ = 45.92 mm ^−1^ s^−1^, 1.5 T). The *r*_2_/*r*_1_ value of 6.103 indicated the feasibility of PDAs@CP_3_‐DOX for *T*_1_–*T*_2_ dual mode MR contrast enhancement. Upon 808 nm laser irradiation, PDAs@CP3‐DOX showed excellent photothermal conversion capacity and NIR laser‐triggered drug release behavior, consequently resulting in improved tumor cell killing effect.

##### Photodynamic Therapy–PTT

In single PDT, most small molecular photosensitizers have poor water solubility and stability in physiological condition, leading to limited bioavailability and compromised PDT efficacy. PDT–PTT bi‐modal synergistic therapy can be realized by integration of PTCAs and photosensitizers onto one nanoplatform. On one hand, PTT‐induced mild hyperthermia can enhance the tumor cell membrane permeability and thus enhance the cellular endocytosis of photosensitizer‐carried nanocarriers. On the other hand, the mild hyperthermia can accelerate the blood flow and improve tumor oxygenation, which is beneficial to enhance the oxygen‐dependent PDT effect. This hyperthermia‐enhanced intracellular ROS generation mechanism makes PDT–PTT synergistic therapy promising against tumors.

For instance, encouraged by the reactivity of the amino groups on MelNPs toward carboxyl groups containing molecules via carbodiimide reaction, chlorin e6 (Ce6) was covalently conjugated onto PDA NPs to achieve PTT–PDT combination therapy.^30^ The exact loading amount of Ce6 on PDA NPs could be facilely controlled and the photostability of Ce6 was enhanced. After intratumoral injection of PDA‐Ce6 NPs in HepG2 tumor‐bearing mice and dual‐wavelength laser irradiation (670 and 808 nm for PDT and PTT, respectively), synergetic PTT–PDT effect was observed. To further potentiate therapeutic safety and efficacy, it remains highly desirable to design intelligent nanoplatforms with controlled photosensitizer release behavior. Based on adenine–thymine complementary base pairing rules, Zhan et al. reported a photothermal‐responsive photosensitizer release nanosystem (A‐PDA = T‐ZnPc).^99^ Following 808 nm laser irradiation, the temperature increase arising from PDA NPs could induce the fracture of adenine–thymine hydrogen bonds and effective release of zinc phthalocyanine. Then, 665 nm laser irradiation was performed for PDT. As expected, the nanoparticles showed excellent biocompatibility and satisfactory therapeutic efficacy in PTT–PDT dual‐modal treatment. A limitation for these two studies was the inconvenience using dual‐wavelength laser irradiation, which increased additional equipment/time cost and might undermine their synergistic interaction. Coactivation of PTCAs and photosensitizers using a single laser irradiation is highly desired, as synchronous PTT–PDT can shun the therapeutic time interval and realize enhanced synergistic efficacy.

**Figure 9:**
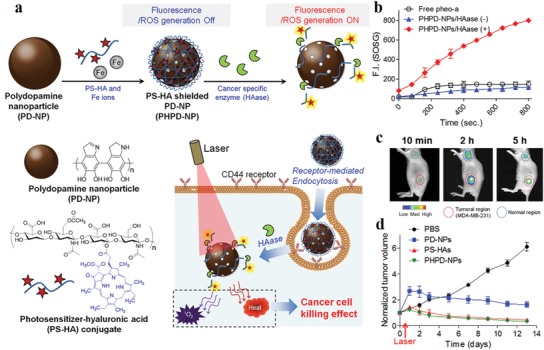
Photosensitizer–hyaluronic acid conjugates shielded PDA NPs for tumor targeted PTT–PDT synergistic therapy. a) Schematic diagram of the synthesis procedure, chemical structure, and theranostic applications of PHPD‐NPs. b) Singlet oxygen generation assessment of PHPD‐NPs in the absence or presence of ten units mL^−1^ hyaluronidase under laser irradiation (*n* = 3). c) In vivo fluorescence images of MDA‐MB‐231 tumor bearing mice as a function of time post subcutaneous injection of PHPD‐NPs (0.1 mg kg^−1^ pheo‐a, upper, normal region; lower, tumoral region). d) Tumor growth curves of MDA‐MB‐231 tumor bearing mice post intratumoral injection with different treatments (*n* = 4). Reproduced with permission.^100^ Copyright 2016, American Chemical Society.

As an indispensable element for PDT, currently available photosensitizers often suffer from undesirable photoactivation by sunlight, poor specificity toward disease sites, and self‐destruction under laser irradiation. To overcome these limitations, Han et al. designed pheophorbide‐a conjugated hyaluronic acid shell shielded PDA NPs (PHPD‐NPs) as a photoactivity controllable nanoplatform for tumor targeted PDT–PTT combination therapy (**Figure** **9**).^100^ The PDA NPs core acted as a quencher for both fluorescence emission and singlet oxygen production of pheophorbide‐a, owing to their electron transfer and energy transfer properties. And pheophorbide‐a conjugated hyaluronic acid shell functioned as a tumor targeting moiety. After receptor‐mediated endocytosis by tumor cells, the hyaluronic acid shell was degraded in response to intracellular hyaluronidase, thereby separating pheophorbide‐a from the PDA NPs core. This led to the complete recovery of fluorescence signal and singlet oxygen production of pheophorbide‐a in the tumor cells. As expected, the as‐prepared PHPD‐NPs exhibited enhanced antitumor activity through synergistic PTT–PDT effect following a single 670 nm laser irradiation. Distinctly, it can make an assertion that the aforementioned surface loading approach inevitably encounters limited surface loading capacity due to the finite specific surface area of MelNPs. Furthermore, the surface properties (e.g., hydrophilicity, surface charge, roughness) of MelNPs may lose with the surface blocking effect of drugs, affecting the blood circulation and cellular endocytosis. On the other hand, hollow nanostructures require complex procedures involving hazardous etching reagents. To address these challenges, Ce6 was encapsulated within the polymer matrix of PDA NPs (PDA‐Ce6 NPs) by a facile two‐step method.^31^ In this strategy, the zwitterionic properties, surface hydrophilicity, and negative surface charge of PDA NPs, as well as their mechanical and structural integrity were retained. The as‐developed PDA‐Ce6 NPs exhibited prolonged Ce6 release profile until day 5 because of π–π stacking interaction. Enhanced cell uptake and PTT–PDT mediated increased cell killing effect against T24 bladder cancer cells were realized following a single 665 nm laser irradiation.

##### Immunotherapy–PTT

As immune microenvironment plays an important role in tumor progression, immunotherapy has emerged as a potent adjuvant therapeutic pillar with proven clinical efficacy.^101^ Emerging nanotechniques based immunotherapy holds great promise in tumor therapeutics. Given that PTT can induce anti‐tumor immune responses,^102^ immunotherapy–PTT can be easily realized on a simple photothermal conversion nanoplatform. In this combined therapy, PTCAs‐produced mild hyperthermia can enhance the intratumoral accumulation, cellular uptake, and release of immunological adjuvants from nanocarriers. Moreover, adaptive immunological responses can be induced to kill the remaining tumor cells in vivo. These superior advantages make immunotherapy–PTT promising in suppressing local primary tumors and distant metastatic tumors, and in preventing tumor relapse.

**Figure 10:**
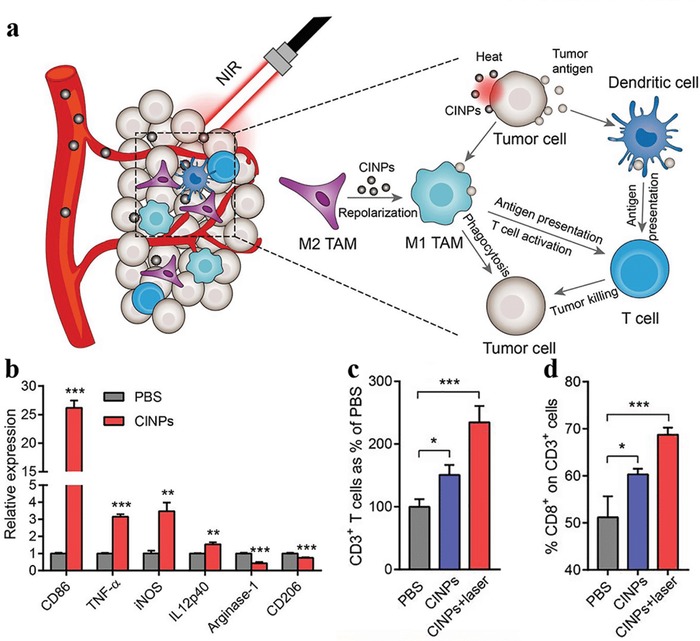
Nanoparticles from cuttlefish ink for tumor immune‐ and photothermal synergistic therapy. a) Schematic diagram of the action mechanism of CINPs for tumor therapy. b) Real‐time qPCR analysis of the gene expression levels in RAW264.7 macrophages. c) Quantitative analysis of T cell infiltration within tumors following different treatments by flow cytometry. d) Quantitative analysis of the percentage of CD8^+^ T cells in CD3^+^ T cells within tumors after receiving different treatments. *p < 0.05, **p < 0.01, ***p < 0.001. Reproduced with permission.^103^ Copyright 2019, American Chemical Society.

Very recently, cuttlefish ink sac extracted nanoparticles (CINPs) comprising melanin, amino acids, and monosaccharides were obtained using a straightforward differential centrifugation method, and their antitumor efficacy by synergizing immunotherapy and PTT were investigated (**Figure** **10**).^103^ The polysaccharides from CINPs could facilitate the repolarization of tumor‐associated macrophages toward anti‐tumor M1‐like phenotype and motivate the infiltration of cytotoxic T cells within the tumor in vivo. The outstanding photothermal effect of melanin from CINPs for tumor could not only be utilized for killing tumor cells directly, but also induce the generation and release of tumor‐associated antigens in situ. As a consequence, by tumor immunotherapy in combination with PTT, these contributors together almost completely inhibited the tumor growth accompanied with enhanced anti‐tumor immune responses and elevated expression levels of proinflammatory cytokines (e.g., IFN‐γ, TNF‐α, IL‐6, IL‐12p40), resulting in significantly suppressed primary tumor growth as well as lung metastasis in CT26 tumor bearing mice. The as‐developed CINPs are promising therapeutic agents attributed to their easy accessibility and versatile functions.

In another work by Ye et al., melanin‐mediated immunotherapy was achieved using an intradermal polymeric microneedle vaccine patch that targets antigen‐presenting cells.^104^ In this vaccine, inactive B16F10 whole tumor lysates containing melanin were encapsulated into a microneedle patch, allowing lysate release in a sustained manner upon insertion into the skin. Upon remotely controllable NIR laser irradiation, local skin temperature rapidly increased toward 42 °C, mediated by the efficient photothermal conversion of melanin in situ at the vaccination site. The hyperthermal‐mimicking microenvironment substantially facilitated the uptake and presentation of tumor‐associated antigens by dendritic cells, leading to enhanced local immune activation through the extensive lymphatic vessel networks. The photo‐responsive combined vaccine increased infiltration of polarized T cells, and elevated expression levels of proinflammatory cytokines (e.g., IFN‐γ, TNF‐α, IL‐6) and immunogenic substrates (e.g., ROS, HSP70, HSP90, antigen adjuvants) that trigger the immune activation. As a result, these B16F10‐specific immunostimulatory effect and immunostimulation responses exerted potent antitumor effect toward both primary tumor and distant tumor, and significantly prolonged the long‐term survival of B16F10 melanoma‐bearing mice. This novel photo‐responsive strategy can be extended to other biological pigments and is also adaptable to other theranostic applications.

##### Chemo‐Radiotherapy

**Figure 11:**
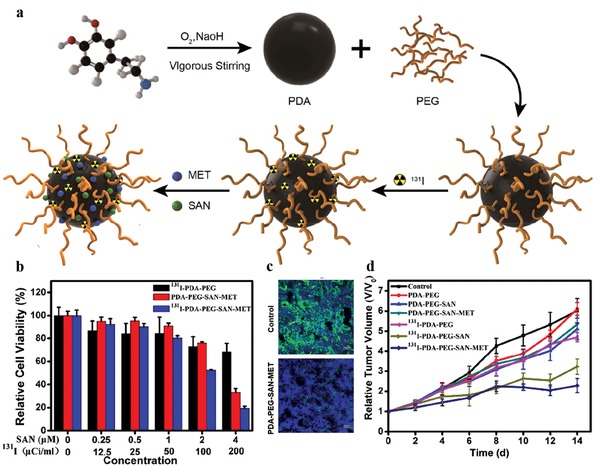
Radionuclide imaging‐guided combined chemo‐radiotherapy using ^131^I‐labeled PDA multifunctional nanocomposites. a) Schematic diagram of the synthesis procedure of ^131^I‐PDA‐PEG‐SAN‐MET nanoparticles. b) Relative viabilities of 4T1 cells after receiving different treatments for 24 h. c) Representative immunofluorescence images of tumor hypoxia at 24 h post injection of ^131^I‐PDA‐PEG‐SAN‐MET. Green and blue represent hypoxia and the nuclei, respectively. d) Tumor growth curves of 4T1 tumor bearing mice with different treatments (*n* = 5). Abbreviations: SAN, sanguinarine; MET, metformin. Reproduced with permission.[qv: 63a] Copyright 2018, Elsevier.

Synergistic chemo‐RT represents one of the most prevalent combination treatment protocols in clinic. The mechanism is related to the action of radiosensitizing components, which can increase the sensitivity of tumor cells to radiation via diverse pathways. For instance, tumor cells can be selectively arrested in G2/M phase by some drugs under X‐ray exposure, resulting in elevated DNA damage. On the basis of the Compton scattering effect, some high‐Z heavy metal elements can be chelated onto MelNPs as effective radiosensitizers for improved dose‐amplification effect. More recently, SAN and metformin (MET) co‐loaded, radionuclide ^131^I labeled, and PEGylated PDA NPs (^131^I‐PDA‐PEG‐SAN‐MET) were developed for radionuclide imaging‐guided chemo‐radioisotope combination therapy (**Figure** **11**).[qv: 63a] The loaded sanguinarine and metformin could suppress the tumor growth via inducing tumor cell apoptosis and alleviating tumor hypoxia, respectively. The labeled ^131^I enabled gamma imaging and radioisotope therapy. More interestingly, the increased tumor oxygenation by metformin could act as a radiosensitizer to overcome hypoxia‐associated radioresistance. As a consequence, the nanocomposites showed superior therapeutic outcome compared with the respective monotherapy. Likewise, radionuclide ^131^I and DOX co‐loaded and PEGylated PDA NPs (^131^I‐PDA‐PEG/DOX) were also reported to realize tumor chemo‐radiotherapy.^62^ In another study by Yi et al., biomimetic copper sulfide nanoparticles were fabricated using melanin as the stabilizing agent and modified with PEG, and then DOX was loaded to obtain CuS@Melanin‐PEG/DOX.^105^ Interestingly, X‐ray ionizing radiation was found to cause increased cellular endocytosis and reduced efflux of various nanoparticles by tumor cells, attributed to the selective cell arrest in G2/M phase and elevated caveolin‐1 levels. Such a phenomenon did not occur for small molecular DOX. As a result, CuS@Melanin‐PEG showed excellent radiosensitization ability to enhance X‐ray‐mediated cell apoptosis, eventually leading to substantially synergistic antitumor performance. This synergistic mechanism would provide an insight into nanoplatform‐based combined chemo‐radiotherapy.

##### Photodynamic Therapy–Radiotherapy

ROS generation is known to be involved in the therapeutic mechanisms of both PDT and RT. ROS (mainly singlet oxygen) is produced via photochemical reaction upon laser irradiation for the former, and ROS (mainly reactive free radicals) is produced upon X‐ray radiation for the latter. Since nuclear deoxyribonucleic acid (DNA) is one of their common targets, there may exist synergistic interactions in cell‐killing between these two therapeutic modalities. By means of this, the possible DNA repair can be effectively inhibited, resulting permanent DNA damage and thus enhanced therapeutic effect. More importantly, the synergistic effect can significantly reduce the laser power density and X‐ray radiation dosage to minimize the adverse effects.

In a work by Yang and co‐workers, PEGylated PDA NPs were co‐loaded with chlorine6 and curcumin (PDA‐PEG/Cur/Ce6 NPs) for combined PDT‐radiotherapy of cancer.^106^ In this nanostructure, the loading efficiency of Ce6 and curcumin could reach 30% and 15%, respectively, and both of them showed a pH‐dependent release behavior. The loaded Ce6 could generate singlet oxygen under NIR laser irradiation for PDT, and curcumin could serve as a radiosensitizer under X‐ray exposure for enhanced RT. After 2 h post intratumoral injection and sufficient diffusion, A549 tumor‐bearing mice were first exposed to 660 nm laser (5 mW cm^−2^, 1 h) for PDT and sequentially exposed to 6 Gy X‐ray for RT. As expected, PDA‐PEG/Cur/Ce6 NPs significantly suppressed the A549 tumor growth, in which the antitumor effect was obviously higher over single PDT and single RT, indicating the remarkable synergistic PDT‐RT effect. This work provides a paradigm of MelNP‐based nanoagents for combined PDT‐RT. Notably, as the work mechanisms of both PDT and RT are oxygen‐involved, the time window between PDT and RT should be well‐explored to guarantee sufficient tumor oxygen. Furthermore, novel oxygen‐carried nanocomposites may be a direction for overcoming tumor hypoxia‐associated resistance and elevated PDT‐RT effect. As DNA locates in the cell nucleus, nucleus‐targeted modules can be functionalized onto MelNPs for enhanced intranuclear radiosensitization efficacy.

##### Anti‐Inflammatory Therapy–PTT

Numerous evidences have shown a close relation between inflammation and a series of cancer events, such as cancer onset, progression, metastasis, and unfavorable prognosis. This is because the inflammatory microenvironment can provide susceptive conditions for epithelial–mesenchymal transition of cancer cells, invasion, and metastasis.^107^ Furthermore, inflammation response induced by PTT may have disadvantageous influence on tumor elimination. In a recent study, an anti‐inflammatory and tumor‐targeted photothermal nanoagent (pDA@aspirin@CM) was presented, by combining PDA nanospheres, anti‐inflammatory drug acetylsalicylic acid (aspirin), and cancer cell membrane coating.^108^ In this design, pDA nanospheres could effectively kill cancer cells via NIR laser mediated photothermal effect, aspirin could suppress PTT‐induced inflammation response via down‐regulation the levels of inflammatory cytokines (e.g., IL‐6 and TNF‐α), and the 4T1 cancer cell membrane coating could provide homologous tumor targeting, respectively. Using this biomimetic PTT strategy, complete tumor thermal ablation was achieved in 4T1 tumor‐bearing mice. This anti‐inflammatory photothermal approach could expedite the next generation of high‐performance agents for improved anti‐tumor effect.

##### Chemotherapy–Microwave Thermal Therapy

As local temperature rise is involved in PTT and MWTT, the advantages of chemotherapy–PTT can be also applied equally to combined chemotherapy–MWTT. Owing to the deep tissue penetration of microwave, chemotherapy–MWTT can be implemented in deep‐seated tumors. Taking the advantages of multifunctionality of MelNPs, Tang et al. reported DOX‐loaded and ionic liquid‐encapsulated hollow PDA nanocomposites (IL‐PDA‐DOX) for tumor chemotherapy–MWTT.^109^ In this design, hollow PDA NPs were first fabricated by template method, then DOX and 1‐butyl‐3‐methylimidazolium hexafluorophosphate were sequentially loaded under vacuum condition and violent stirring. The loading ratio of ionic liquids and DOX were calculated to be 22.8% and 10.79%, respectively. The nanocomposites exhibited wonderful microwave sensitization effect even under relatively low microwave power, owing to the contribution of ionic liquids. After microwave irradiation in the IL‐PDA‐DOX nanocomposites, splendid antitumor effect was observed in H22 tumor‐bearing mice, the tumor was completely eliminated without regrowth during an observation period of 16 days. The therapeutic efficacy was remarkably higher compared with different control groups, indicating the synergistic chemotherapy–MWTT effect. This work provided a novel combined therapy strategy against tumors. To further enhance the therapeutic efficacy, targeting modules can be functionalized onto MelNPs for tumor‐specific delivery. Furthermore, the appropriate temperature range for maximized drug release should be well explored.

### 4.4 Other Applications

#### 4.4.1 Antioxidative and Anti‐Inflammatory Therapy

Antioxidative therapy is gaining increasing attention as an efficient therapeutic approach against a myriad of RONS involved diseases, as elevated RONS levels induced oxidative stress is a pivotal mechanism implicated in the progression of various pathological processes.^110^ However, the majority of current single component‐based antioxidants simply exhibit scavenging specificity toward given RONS, and thus show insufficient scavenging against multiple persistent RONS simultaneously generated in disease processes.^111^ On the other hand, multiple component‐based antioxidative defense systems usually require complicated and time‐consuming fabrication processes, and inevitably bring undesirable residue and potential adverse reactions. Furthermore, natural antioxidant enzymes often suffer from low stability, and difficulty of reuse and long‐term storing. These limitations together hamper their practical biomedical applications and clinical translation. In this regard, ideal antioxidants must meet the following criteria: i) robust and broad‐spectrum scavenging of multiple pathogenic RONS; ii) highly stable defensive ability against RONS‐mediated oxidative damage and inflammatory response; iii) satisfactory biocompatibility and biodegradability.

**Figure 12:**
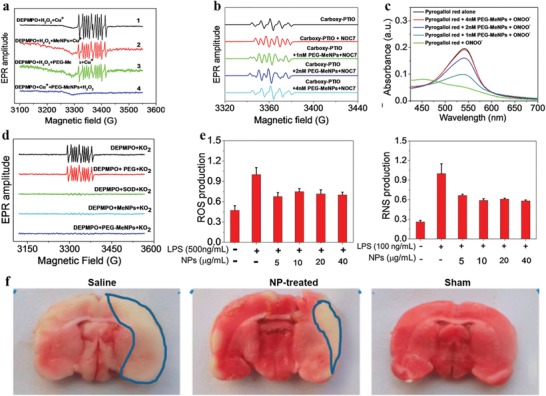
Multi‐antioxidative mechanisms of MelNPs and their therapeutic applications in ischemic stroke. a) EPR spectra for detection of hydroxyl radical. b) EPR spectra for detection of nitric oxide radical. c) UV–vis absorption spectra for detection of peroxynitrite. d) EPR spectra for detection of superoxide anion radical. e) Reactive oxygen (left) and nitrogen (right) species in lipopolysaccharide‐stimulated macrophages. f) Representative digital photographs of 2,3,5‐triphenyltetrazolium chloride‐stained brain slices from different groups. Reproduced with permission.^34^ Copyright 2017, American Chemical Society.

The unique radical scavenging ability of melanins and their derivatives render them promising as natural antioxidative platforms, owing to the abundant reductive functional moieties such as amine, imine and catechol.^33^, ^112^ For the first time, Liu et al. found that the bioinspired melanin nanoparticles (MeNPs) could protect the ischemic brain tissues from oxidative damage in an ischemic stroke rat model (**Figure** **12**).^34^ The PEG‐MeNPs exhibited potent and broad antioxidative activities against primary and secondary RONS, including hydrogen peroxide, superoxide anion, hydroxyl radical, peroxynitrite, and nitric oxide radical. Additionally, PEG‐MeNPs effectively inhibited RONS‐mediated inflammatory responses, confirmed by reduced expression levels of inflammatory factors and cytokines. This study improves our understanding of the broad‐spectrum antioxidative effects and mechanisms of melanin, and lays the foundation for further designing efficient and safe antioxidant defense platforms against many other RONS associated diseases. For instance, ultrasmall Mn^2+^‐chelated melanin nanoparticles (MMPP) were reported as a natural antioxidant defense nanoplatform, and they demonstrated preferential renal accumulation and robust antioxidative protection response in treating murine acute kidney injury.^65^ In another study, Zhao et al. described that PDA NPs could eliminate ROS for treating acute inflammation‐induced injury.^113^ After a single dose treatment, PDA NPs exhibited significant anti‐inflammation effect in mouse models including acute lung injury and acute peritonitis. The anti‐inflammation effect mainly manifested as reduced ROS generation and proinflammatory factors levels, suppressed neutrophil infiltration, and attenuated tissue damage. Similarly, Bao et al. evaluated the efficacy of PDA NPs as broad‐spectrum ROS scavengers in a mouse periodontitis model.^114^ The PDA NPs exhibited excellent scavenging activities against toxic hydroxyl radicals and superoxide radicals in vitro. Cell experiments showed that PDA NPs could protect human gingival epithelial cells from oxidative stress and inflammation response, owing to their ROS scavenging and anti‐inflammatory activity. After post‐subgingival injection in vivo, the oxidative stress induced inflammation reaction was effectively suppressed without any side effect. This was attributed to the improved local periodontal microenvironment by efficient ROS removal. The results suggest the potential of PDA NPs as an anti‐inflammatory nanoplatform.

#### 4.4.2 Antibacterial Infections

Antibiotics treatment has achieved exciting successes against various bacterial infections during the past couple of decades. Dismayingly, the efficacy of currently available antibiotics is dramatically diminishing ascribed to the long‐standing immoderate abuse of antibiotics and increasing emergence of antibiotic resistance.^115^ For instance, methicillin‐resistant *Staphylococcus aureus* (MRSA) strains infection and associated complications (e.g., sepsis and acute endocarditis) represent alarming challenges in clinical practice. Novel potent antibacterial materials are urgently warranted to effectively eliminate drug‐resistant bacterial infections.

Metals species such as silver and copper are well known to possess antibacterial properties mediated by specific mechanisms. For enhanced activity, these metals require stabilization by various substances to render them as antibacterial nanoagents. However, the process is often quite complicated and time consuming. Inspired by the high metal ions chelating capability of MelNPs, Yeroslavsky et al. reported a facile one‐pot oil–water sonochemical approach to prepare Cu‐containing PDA capsules, demonstrating rapid (15 min) and strong (99.9%) bactericidal activity.^116^ Subsequently, this group adopted the same procedure to fabricate core/shell Ag‐, Cu‐, and hybrid Cu/Ag‐based PDA NPs.^117^ The metal‐incorporated PDA NPs exhibited broad‐spectrum and more potent antibacterial activities against four types of tested bacterial strains, compared with commercially available silver nanoparticles. Interestingly, they could effectively prevent the biofilm formation and eradicate already formed biofilms that are incompetent by common antibiotics. This was attributed to the combined antibacterial mechanisms of these two metals. The generated semiquinone and reactive oxygen species by PDA also contributed to the antimicrobial activity. This simple and environmentally friendly fabrication procedure holds promise in the development of metals incorporated nanoplatforms for antibacterial applications. Notably, metal‐containing nanoagents may encounter nonspecific biological toxicity when megadose administration and long‐term retention in vivo.^118^ This inspires the development of metal‐free antibacterial agents. Recently, nitric oxide gas molecules mediated antibacterial strategy has received much attention, because its radicals and byproducts can induce oxidative and nitrosative stresses in various kinds of bacterial species, leading to disrupted and dysfunctional bacteria membrane. In view of this, diazeniumdiolate functionalized hollow PDA NPs (PDA‐NO HNPs) were reported as efficient nitric oxide delivery systems and antibacterial agents.^119^ The hollow PDA NPs showed high nitric oxide adsorption upon exposure to high pressure nitric oxide gas under basic conditions. The as‐prepared PDA‐NO HNPs exhibited negligible cytotoxicity, and sufficient nitric oxide release profile for killing Gram‐negative bacteria.

**Figure 13:**
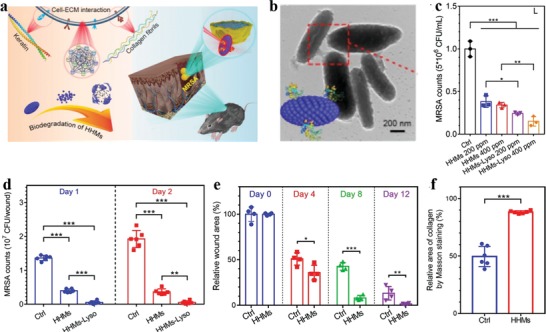
Human hair‐derived melanosome nanostructures for combating MRSA infection and accelerated tissue repair. a) Schematic illustration of HHMs‐Lyso for infection eradication and tissue repair applications. b) TEM images of HHMs‐Lyso. c) Antibacterial activity of HHMs and HHMs‐Lyso upon 808 nm laser irradiation (*n* = 3). d) Quantitative analysis of bacterial counts by spread plate method in MRSA‐infected wounds on day 1 and day 2 (*n* = 6). e) Quantitative analysis of the wound healing profiles over time (*n* = 4). f) Quantitative collagen deposition analysis using Masson's trichrome staining (*n* = 6). Abbreviations: MRSA, methicillin‐resistant staphylococcus aureus; Lyso, lysozyme; HHMs, human hair‐melanosome derivatives. Reproduced with permission.^19^ Copyright 2019, American Chemical Society.

Apart from tumor thermal ablation applications, PTT has also emerged as a potential approach to effectively disrupt the bacterial membrane and kill bacteria.^120^ This physical hyperthermia possesses broad‐spectrum antibacterial capability in a rapid and efficient manner, as well as minimal damage to healthy tissues. Synergistic treatment combining hyperthermia and antibacterial agents is an encouraging strategy to strengthen the antibacterial performance. In a very recent work, natural mulberry‐shaped human hair‐derived melanosome (HHMs) nanostructures composed of structural keratins and functional melanins were easily extracted by using a straightforward low‐temperature alkali heating procedure (**Figure** **13**).^19^ Then, positively charged lysozyme, an antimicrobial glycoside‐hydrolase enzyme, was electrostatically absorbed on the surface of negatively charged HHMs to obtain HHMs‐Lyso nanocomposites. The as‐prepared HHMs‐Lyso nanostructures exhibited a high eradication efficacy up to 97.19 ± 2.39% against MRSA infection in vivo, even higher than that of positive control vancomycin (87.75 ± 2.02%). This was attributed to the synergistic action of photothermal effect by melanins and “lysozyme‐assisted anti‐infection.” Notably, the upregulation of collagen alpha chain proteins within the extracellular matrix by keratins, mediated by the “protein digestion and absorption” signaling pathway, accelerated the tissue reconstruction and wound closure processes in a MRSA wound mouse model. Encouragingly, HHMs‐Lyso nanocomposites showed excellent biocompatibility and a proper biodegradation behavior in the presence of oxidizing agents. This work may inspire innovative personalized therapy strategies for bacteria elimination and synchronous tissue reconstruction by using patients′ own hair.

Despite that various antibacterial nanoagents have been developed, effective treatment of bacterial infections at the site with high shear forces (e.g., bloodstream, urinary tract, cornea surface) remains challenging. This is due to the fact that high shear forces not only can encourage bacterial adhesion by enhancing the receptor‐specific bacteria–host interactions, but also inhibit localized antibiotic retention following bacterial colonization.^121^ Along this line, bioadhesive ciprofloxacin‐loaded nanoparticle–hydrogel (NP–gel) hybrid were reported for localized antibiotic delivery under high shear force conditions.^122^ In this formulation, ciprofloxacin was encapsulated into polymeric nanoparticles using a double emulsion solvent evaporation technique, and then the nanoparticles were embedded into dopamine methacrylamide integrated hydrogel network. The as‐prepared NP–gel system showed a controlled and sustained release profile of loaded Cipro molecules, whereas free Cipro exhibited a burst release behavior in the control group. Compared to the nonadhesive NP–gel, the bioadhesive NP–gel system exhibited much more superior adhesion and antibiotic retention performance under high shear forces on three types of representative biological surfaces, including an *E. coli* bacterial film, a HEK 293T cell monolayer, as well as shaved mouse skin tissue. The Cipro‐loaded NP–gel effectively inhibited the bacterial film formation under flow conditions in vitro. With tunable adhesion strength and viscoelasticity, the tissue‐adhesive NP–gel system holds great potential to serve as a safe, prolonged, and enhanced localized delivery platform that withstands high shear forces.

#### 4.4.3 Neuroprotection in Parkinson's Disease

**Figure 14:**
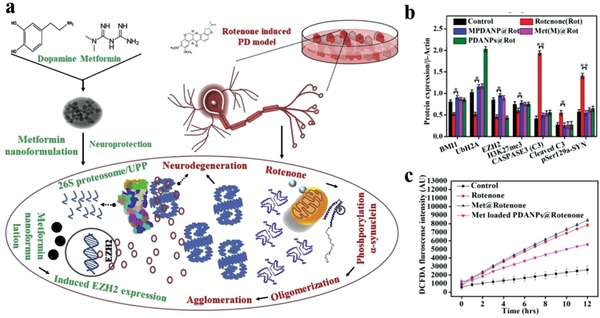
Neuroprotection and anti‐Parkinsonism effect of metformin loaded PDA NPs. a) Schematic diagram of the neuroprotective mechanisms of Met loaded PDANPs. b) Quantitative protein analysis by western immunoblotting. c) Anti‐inflammatory response analysis of Met loaded PDANPs by reactive oxygen species quenching detection. Abbreviations: MET, metformin. Reproduced with permission.^123^ Copyright 2019, Elsevier.

Posttranslational modification and aggregation of phospho‐serine 129 α‐synuclein, mitochondrial oxidative stress, and gradual dopaminergic neuron degeneration are hallmarks involved in the course of Parkinson's disease progression, leading to the formation of Lewy bodies and synucleinnopathy, aggressive phenotype, and depressed dopamine level in the substantia nigra, respectively. Despite that metformin has emerged as a potent neuroprotective agent against Parkinsonism via several mechanisms, it possesses limited bioavailability and potential risk of lactic acidosis. To overcome these challenges, metformin encapsulated PDA NPs were synthesized and applied in a Parkinson's disease model (**Figure** **14**).^123^ The metformin encapsulated PDA NPs demonstrated excellent neuroprotective efficacy, mediated by upregulation of Enhancer of zeste homolog 2 expression, degradation of phospho‐serine 129 α‐synuclein, reduction in oxidative stress, suppression of apoptosis and inflammatory response. Importantly, the PDA could serve as antioxidant and dopamine replenisher in addition to a nanocarrier for improving bioavailability, which could further enhance the neuroprotective efficiency. This as‐developed nanoformulation holds neuroprotective potentiality in other types of neurodegenerative disorders.

Dopaminergic neurons degeneration induced neurotransmitter dopamine loss is a key character in Parkinson's disease.^124^ Replenishment of missing dopamine is regarded as an effective approach to relieve uncontrolled gait imbalance and behavioral deficits. However, dopamine is an impermeable agent across the blood brain barrier, and the side effects of frontline drugs in clinic outweigh their therapeutic benefits.^125^ To address these issues, neurotransmitter dopamine‐loaded poly(lactic‐*co*‐glycolic acid) nanoparticles (DA NPs) were developed for delivery of dopamine across the blood brain barrier in 6‐hydroxydopamine‐induced Parkinsonian rats.^126^ The as‐prepared DA NPs reduced cytotoxicity, dopamine autoxidation and plasma clearance in comparison to bulk dopamine. After intravenous injection, DA NPs effectively penetrated the blood brain barrier and localized at the brain striatum and the substantia nigra, increased and maintained the striatal dopamine levels through sustained and slow dopamine release, decreased dopamine‐D2 receptor supersensitivity, and eventually reversed neurochemical and neurobehavioral abnormalities. Interestingly, this nanoformulation did not bring about additional ROS and quinone adducts generation, dopaminergic neuron degeneration, mitochondrial damage and changes in ultrastructure. Moreover, neither detrimental cardiovascular effect nor other noticeable toxicity was observed, suggesting the safety and effectivity of this delivery nanoplatform.

#### 4.4.4 Wound Healing and Medical Bioadhesives

**Figure 15:**
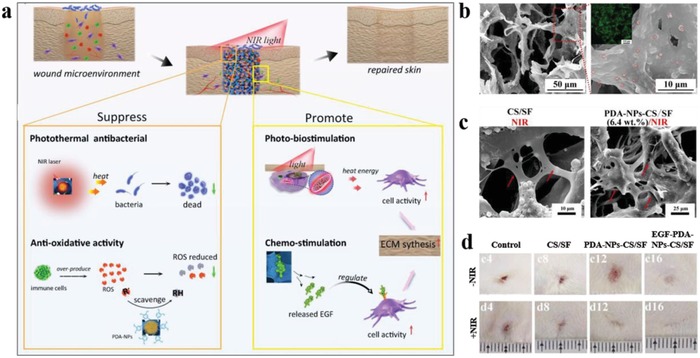
Multifunctional mussel‐inspired chitosan/silk fibroin cryogels for promoting wound regeneration and suppressing bacterial invasion. a) Schematic diagram of the action mechanisms of PDA‐NPs‐CS/SF cryogels. b) SEM images of PDA‐NPs‐CS/SF cryogels (CS/SF = 2:1, 6.4 wt% PDA‐NPs). c) SEM images of fibroblasts cultured on cryogels with or without laser treatment. d) Representative photographs of defects on day 21 after receiving different treatments. Abbreviations: CS, chitosan; SF, silk fibroin. Reproduced with permission.^128^ Copyright 2019, Royal Society of Chemistry.

Skin wound healing process is a dynamic and considerably complex course involving a series of progressive pathological events, including potential bacterial invasion, inflammatory response, and tissue regeneration and remodeling at the wound site.^127^ The complex mechanisms motivate scientists to exploit multifunctional skin substitutes with multiple biostimulation capabilities toward the unique wound healing microenvironment during wound healing. In a recent work by Han et al., mussel‐inspired PDA NPs incorporated chitosan/silk fibroin (PDA‐NPs‐CS/SF) cryogels were fabricated for wound microenvironment regulation and accelerating skin tissue regeneration (**Figure** **15**).^128^ The unique design of cryogels enables them to regulate multiple aspects of the healing environment, including interconnected microporous networks for cell recruitment, proliferation and tissue ingrowth, antioxidative activity of PDA NPs for suppressing inflammatory response, and chitosan‐ and photothermal‐mediated antibacterial activity for inhibiting bacterial invasion. As expected, complete skin‐thickness wound healing was achieved, mediated by bio‐chemo‐photothermal combined treatment capacity of cryogels. In another study, tetracycline hydrochloride loaded PDA NPs/cellulose nanofibrils (TOCNFs) hydrogels were developed via ion‐crosslinking method.^129^ The as‐developed PDA/TOCNFs hydrogel demonstrated pH/NIR laser responsive drug release and enhanced wound healing. These evidences indicate that the multifunctional melanin‐like nanomaterials represent promising biopolymer‐based dressings for wound healing applications.

In the field of bioadhesives, implantable biomaterials require sufficient and intimate adhesive interactions with the substrate tissues in vivo to fulfill their functions.^130^ Robust bioadhesivity is crucial to prevent undesired material displacement from the implant site.^131^ Evidences suggested that the functionalization using catechol‐containing molecules could enhance cohesive and adhesive strength to biological tissues in moist environment in vivo, through easy oxidization of catechol into *o*‐quinone and then crosslinking reactions.^132^ To maximize the reactive catechol compounds, MelNPs functionalization of biomaterials may be an alternative approach owing to their high specific surface area. In this regard, Scognamiglio et al. designed a MNPs‐based adhesive interface homogeneously spreading on the surface of implantable polysaccharidic surgical membranes.^133^ The thickness of the MNPs continuous coating layer was in the range 30–50 µm and it enhanced bioadhesion of surgical membranes to moist intestinal serosa. This may be benefitted from that the abundant reactive *o*‐quinones facilitate the formation of covalent bonds with amines in biological tissues.^134^ Additionally, the MNPs exhibited a dose‐dependent antibacterial effect against *E. coli* and *S. aureus* strains. These findings will shed new light on devising innovative MelNP‐based adhesive nanosystems for specific medical needs.

#### 4.4.5 Irradiation Protection

The constitutively synthetic melanin in many fungal species can protect them from various types of cosmic and terrestrial ionizing radiation such as ultraviolet and solar.^135^ Most dramatically, melanized fungal species could colonize in remarkably extreme environments with elevated radiation levels (e.g., destroyed chernobyl reactor, nuclear reactor cooling pools.^136^ This “radiotropism” phenomenon and plenty of laboratory evidences suggest a pivotal role of melanin in protection from ionizing radiation. Several attempts have been made to elucidate the related radioprotective mechanisms of melanin. For example, Dadachova's group concluded that the radiation shielding effect resulted from gradual dissipation of high‐energy recoil electrons, due to the enriched aromatic oligomers containing multiple π‐electron systems on melanins.^137^ This can prevent secondary ionization damage and destructive free radical species generation. This group also proposed that the radioprotective property of fungal melanin was considered to be related to its physical shielding, chemical composition, free radical removal and spherical spatial arrangement.^138^ In another study, the interaction between gamma radiation and melanin was capable of altering the oxidation–reduction potential of melanin.^139^ Furthermore, free radical scavenging by water radiolysis may also contributed to this property, decreasing the clastogenic effects of ionizing radiation.^140^

The radioprotective property of melanin motivates us to exploit MelNP‐based nanoagents for radioprotective applications. The high radiosensitivity of hematopoietic system is a dose‐limiting factor for radioimmunotherapy and external beam RT. Doses in excess of 200 cGy can lead to severe acute and long‐term hematologic toxicity. Ionizing irradiation produces reactive oxygen species and free radicals during radiotherapy, which attack various critical cellular macromolecules and induce cell damage and dysfunction.^141^ Protection of bone marrow from myelotoxic effects during radiotherapy can reduce radiotoxicity, allowing for higher radiation doses and thus increased therapeutic efficacy. Inspired by the ^99m^Tc‐labeled sulfur “nano‐colloid” for bone marrow imaging in nuclear medicine practice, Schweitzer et al. reported melanin‐covered nanoparticles for melanin delivery into the bone marrow.^142^ 125cGy of whole‐body irradiation and 1 mCi of radioimmunotherapy were performed in healthy CD‐1 mice and A2058 human metastatic melanoma bearing mice, respectively. A single dose of 50 mg kg^−1^ nanoparticles showed protection of bone marrow hematopoietic cells against radiation fluxes, presenting as decreased counts of white blood cells and platelets. Similarly, synthesized melanin nanoparticles were proved to be effective radioprotectors against whole‐body irradiation induced damages via hematopoietic tissue restoration in mice.[qv: 7a] In another study, Kunwar et al. isolated eumelanin from the fungus *Gliocephalotrichum simplex*. By exposure to lethal 7 Gy whole‐body irradiation, a dose of 50 mg kg^−1^ melanin exhibited both prophylactic and mitigatory activities in Balb/c mice. The 30‐day survival of mice was increased by 100% and 60%, respectively.^39^ The probable whole‐body irradiation protection mechanisms by fungal melanin appeared to include prosurvival ERK pathway modulation, oxidative stress prevention, and immunomodulation. The robust radioprotection property and easy accessibility of melanin highlight the future clinical implication of MelNPs for preventing radiotoxicity in the radiation oncology field and exploiting novel biomimetic melanin‐coated medical radioprotective equipment in nuclear emergencies.

#### 4.4.6 Biosensing

Aberrant expression levels of specific key molecules and biomarkers are closely associated with disease initiation, progression and prognosis. Ultrasensitive detection of their ultratrace levels is of great significance for early accurate diagnosis, monitoring of crucial events in disease evolvement, and clinical decision making. Conventional detection methods based on the historrhexis and cell lysates are complicated and time‐consuming, and often suffer from low sensitivity and interference from other biomolecules. It is highly desirable to develop rapid, simple, and cost‐effective determination platforms with high sensitivity and selectivity.

Among various immunoassay approaches, amperometric immunoassay is highly attractive owing to its distinct superiorities including easy fabrication, rapid determination, high sensitivity and low cost. For instance, based on a peptide‐cleavage‐based electrochemical biosensor, Zheng and Ma described a dual‐reaction amplified sensitivity strategy for ultrasensitive matrix metalloproteinase‐7 detection.^143^ Palladium–PDA nanocomposites acted as dual‐catalysts to trigger catalytic precipitation reaction and Fenton‐like reaction, leading to dramatically enhanced current signal differences for sensitivity amplification. By integration of Au‐rGO/MB‐SA substrates with palladium‐based catalytic probes onto a peptide‐cleavage‐based electrochemical biosensor, ultrasensitive detection performance of matrix metalloproteinase‐7 was achieved, presenting as broad and excellent linearity ranging from 10 fg mL^−1^ to 10 ng mL^−1^, with a detection limit as low as 3.1 fg mL^−1^. This as‐proposed strategy represents a promising nanoplatform which can be easily expanded for detection of many other proteases. In another work, this group developed a label‐free amperometric immunoassay platform for ultrasensitive determination of carcinoembryonic antigen by using PDA‐Pb^2+^ redox system deposited and chitosan‐gold nanocomposites coated glassy carbon electrode.^144^ Owing to the large specific surface area, excellent electroconductivity and strong current response of the platform, the immunosensor showed a wide linear range from 1 fg mL^−1^ to 100 ng mL^−1^, with an ultralow detection limit of 0.26 fg mL^−1^.

**Figure 16:**
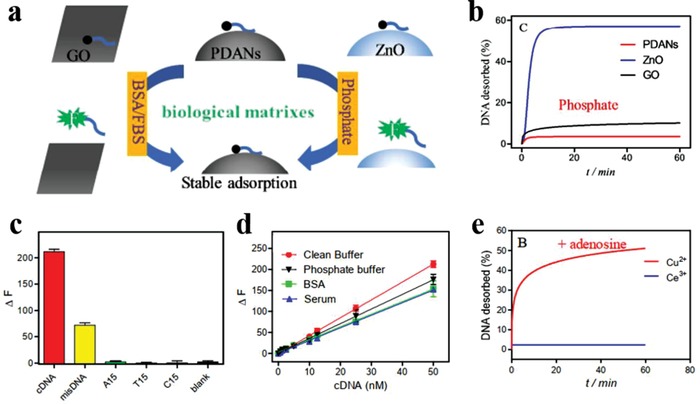
Metal coordination mediated bioorthogonal DNA/PDAN probes for sensitive and selective biosensing applications. a) Schematic diagram of PDAN, zinc oxide and graphene oxide probes in complex biological matrixes which potentially involves nonspecific probe displacement. b) DNA fluorescence recovery profiles of PDAN, zinc oxide and graphene oxide probes after adding 10 ×10^−3^ m phosphate. c) Specificity of cDNA detection by DNA/PDAN probes with different DNA sequences at a concentration of 50 ×10^−9^ m. d) Performance of DNA/PDAN probes for target DNA detection in various biological buffers. e) Desorption kinetics of DNA from 50 ×10^−6^ m Ce^3+^ or Cu^2+^ adsorbed PDANs in presence of 10 × 10^−3^ m adenosine. Reproduced with permission.^145^ Copyright 2018, American Chemical Society.

Selective DNA detection in complex biological fluids remains challenging, because nonspecific DNA displacement by various competitive biomolecules often causes false positive signals. Inspired by the high selectivity of bioorthogonal chemistry and metal ion chelation capacity of MelNPs, metal coordination mediated DNA bioorthogonal adsorption on PDA NPs biosensor was reported (**Figure** **16**).^145^ The catechol groups on PDA NPs provided metal coordination sites for divalent calcium ions, which produced an attractive adsorption force to bridge DNA onto PDA NPs. Such a distinct bioorthogonal adsorption mechanism could resist nonspecific DNA displacement by various biomolecules, showing superior DNA detection performance compared to conventional zinc oxide and graphene oxide nanomaterials. The DNA/PDA NPs nanoprobe exhibited an ultralow detection limit of <1 ×10^−9^ mtoward target DNA in various biological samples. In addition, imaging of microRNA‐21 in A549 lung cancer cells and RLE‐6TN normal cells showed high specificity. Considering the critical role of metal coordination in DNA adsorption, the binding sites and affinities can be modulated by using different metal ions (e.g., Ce^3+^, Cu^2+^). This work indicates the potential of DNA/PDA NPs nanoprobe as a robust DNA detection platform for biological analysis applications.

Fluorescence biosensors, on the basis of Förster resonance energy transfer, own advantages including high sensitivity, in situ imaging, and operational convenience. MelNPs are well known to possess excellent fluorescence quenching ability toward various kinds of fluorophores,^146^ possibly attributed to the excited electrons capture capacity by quinone residues.^147^ In view of this, MelNPs‐based fluorescence biosensors hold great promise for highly sensitive and selective biosensing applications, such as detection and imaging of microRNA and hydrogen peroxide,^148^ DNA and thrombin,^146^, ^149^ adenosine triphosphate,^150^ reactive oxygen species,^151^ and cancer cells.^152^ For example, in a study by Ma et al., FITC‐labeled single strand DNA (ssDNA) loaded PDA NPs were presented as a fluorescent “turn‐on” platform for selective and sensitive sensing of antioxidants in complex biological fluids.^153^ When in the absence of antioxidants, PDA NPs were formed by spontaneous oxidation–polymerization of dopamine monomers. Then ssDNA was adsorbed on their surface with strong affinity, attributed to π–π stacking interaction between nucleobases and aromatic groups. The fluorescence of FITC‐ssDNA was effectively quenched via Förster resonance energy transfer, presenting as the fluorescence “off” state. When in the presence of antioxidants (e.g., ascorbic acid, glutathione, homocysteine, cysteine), the oxidation–polymerization of dopamine was effectively inhibited, leading to the desorption of FTIC‐ssDNA from the surface and efficient fluorescence recovery, presenting as the fluorescence “on” state. This fluorescent “turn on” strategy allowed the sensitive and selective detection of antioxidants, avoiding interferences of various amino acids and metal ions which commonly existing in complex biological fluids. In addition, by integration with in vivo microdialysis technique, continuous monitor of the dynamic variation of striatum antioxidants in cerebrospinal dialysates was achieved in a rat cerebral ischemia model. The present study provides a convenient and broadly applicable method in biochemical and brain chemistry investigations.

## 5 Concluding Remarks and Perspectives

In summary, this feature article gives a systematical, comprehensive, detailed and well‐organized description of recent advancements of bioinspired MelNPs regarding diverse biomedical applications during the past decade. This panoramic Review is expected to bolster our understanding of melanin‐derived nanomaterials and motivate further optimization design of tailorable and marketable multifunctional nanoplatforms in biomedicine. Despite that numerous evidences have suggested MelNPs as a robust and versatile “all in one” nanoplatform with extraordinary promise, there are still some unsolved scientific challenges and technical issues need to be taken seriously prior to eventual clinical translation.

For nanomaterial design and optimization: i) More efforts based on experimental and computational approaches should be directed toward the understanding of detailed polymerization mechanisms and formation kinetics involved in MelNPs synthesis, definite chemical structures, and structure–property–function relationships of MelNPs. ii) Innovative characterization technologies are desired to promote the understanding of undesirable side reactions and byproducts during the complicated polymerization processes and avoid them to the most extent. iii) Standardized extraction and purification procedures should be established to obtain natural melanins with unmodified characteristics. Considering the complicated formation kinetics of MelNPs, reaction conditions should be precisely controlled to obtain MelNPs with stable product performance among different batches. More facile, low‐cost, environment friendly and precisely controllable large‐scale fabrication procedures need to be exploited to meet clinical and commercial needs. iv) Given the relatively low NIR absorption of MelNPs compared with traditional gold nanoparticles, advanced computational modeling techniques are required for prediction and better understanding of the structure–property–function relationships of MelNPs. Tailored strategies can help to achieve even better optical properties for theranostic applications. v) MelNPs have a tendency toward aggregation in aqueous solutions owing to their strong adhesive capability and hydrophobic organic compounds, which undoubtedly hampers their long‐term storage, transportation, and future use. More efforts should be devoted to develop novel approaches for controlling their aggregation state and enhancing their solubility in aqueous phase.

For in vivo biomedical applications: i) Despite considered to be biocompatible, the biodistribution pattern, biodegradation process, metabolic pathway, and potential mid‐ and long‐term implication on organs and organisms particularly the immune system remain unclear and require further explorations systematically and thoroughly. The high reactivity of MelNPs is somewhat favorable, but it may bring negative effects when retention in vivo. ii) Despite tremendous efforts and considerable progresses in MelNPs‐assisted tumor theranostics, the uncontrollable pharmacokinetic profiles, dissatisfactory penetration across physiopathologic barriers, and insufficient intratumoral distribution restrict their translation into clinical practice. To overcome these limitations, more specific targeting modules should be excavated and routinely incorporated on the nanoplatforms to realize desirable targeting capacity. Considering the tumor heterogeneity and the unique tumor microenvironment, more sophisticated multifunctional nanoplatforms with multi‐targeting and multi‐stimuli‐responsive activation capacities would help to realize precise and optimal tumor accumulation. iii) On account of the versatile functionality of MelNPs, multi‐modal imaging and multi‐modal therapy could be achieved on one nanoplatform to maximize the theranostic performance. Higher expectations are always accompanied by more strict requirements. More advanced and updated instruments with multiple integrated excitation sources (e.g., PTT, PDT, RT, MWTT) and deeper tissue penetration depth are highly desirable for synchronous multi‐modal therapies and maximized synergistic therapeutic effect, rather than sequential multiple treatments using different machines. iv) Despite impressive advancements, many fundamental aspects of MelNPs remain as scientific gaps and their potential applications will be far beyond what has been reported so far. For instance, by changing the surface potential of MelNPs from negative into positive, siRNA and miRNA can be delivered for gene therapy. Other diverse combinations of different therapeutic modalities can be integrated into MelNPs based nanostructure to achieve tri‐modal synergistic therapy and thus optimal theranostic performance. As dopamine is an essential neurotransmitter, multifunctional MelNPs based nanomedicine against many neurodegenerative diseases can be anticipated. v) Instead of using commercialized tumor cell lines based subcutaneous tumor models, a new generation of deep‐seated orthotopic tumor models based on patient‐derived primary tumor cells should be used in further studies, which is more likely to mimic the physiopathologic characteristics of the tumor more authentically. vi) Regarding easily metastasized tumors, immunotherapy‐based synergistic therapy may be a fascinating option to activate the immune system and eliminate distant metastatic tumor cells. Considering the inherent immunoregulatory capacity of dopamine, collaborative PTT‐immunotherapy could be readily implemented in a simple system. Moreover, prolonged in vivo observation period and comprehensive efficacy evaluation assays should be performed to obtain a credible assessment report.

As a concluding remark, the past decade has witnessed unprecedented strides in the development of multifunctional MelNPs as high‐performance theranostic nanoplatforms. It is indisputable that booming research in MelNPs would make substantial breakthroughs and become commercially practicable in the near future. This motivates further studies through multidisciplinary full collaborations across biologists, physicists, chemists, pharmacologists, materials scientists, and clinicians.

## Conflict of Interest

The authors declare no conflict of interest.
